# Population structure and genetic diversity of invasive Fall Armyworm after 2 years of introduction in India

**DOI:** 10.1038/s41598-021-87414-5

**Published:** 2021-04-08

**Authors:** N. Nayyar, R. G. Gracy, T. R. Ashika, G. Mohan, R. S. Swathi, M. Mohan, M. Chaudhary, N. Bakthavatsalam, T. Venkatesan

**Affiliations:** 1 ICAR-National Bureau of Agricultural Insect Resources, Bellary Road, Hebbal, Bengaluru, 560024 India; 2 CABI, South Asia-India, CG Block, NASC Complex, DP Shastri Marg, PUSA, New Delhi, 110012 India

**Keywords:** Evolutionary genetics, Population genetics, Invasive species

## Abstract

Fall Armyworm (FAW), *Spodoptera frugiperda*, is a polyphagous pest capable of feeding over 80 plant species and was indigenous to the Western Hemisphere. Within a span of 4 years, FAW has established itself throughout most of the regions in Africa and Asia causing significant losses in maize production. Owing to its revamped distribution range, it would be prudent to analyze the ensuing genetic changes and study the emerging phylogeographic patterns across the world. In this regard, we would like to provide a current snapshot of genetic diversity of FAW in India 2 years after the initial introduction and compare it with the worldwide diversity in order to trace the origins and evolutionary trajectories of FAW in India. We have investigated around 190 FAW samples from different regions in India for strain identity and polymorphism analysis on the basis of partial mitochondrial *cytochrome oxidase* I (COI) gene sequences. Apart from the ancestral rice and corn strain haplotype, our study demonstrates the presence of 14 more haplotypes unique to India at a haplotype diversity of 0.356. We were also able to record inter-strain hybrid haplotypes of rice and corn strains in India. Regional heterogeneity within Indian populations seems to be quite low representative of extensive migration of FAW within India. Distribution analysis of pairwise differences and rejection of neutrality tests suggest that the FAW population in India might be undergoing expansion. Our data is consistent with the findings suggesting a recent and common origin for invasive FAW populations in Asia and Africa, and does not indicate multiple introductions to India. This study reports the highest genetic diversity for Indian FAW populations to date and will be useful to track the subsequent evolution of FAW in India. The findings would have important ramifications for FAW behavior and composition throughout the world.

## Introduction

Fall Armyworm (FAW), *Spodoptera frugiperda* (J.E. Smith), is a polyphagous pest native to Western Hemisphere and is capable of feeding over 80 plant species. In 2016, it was first reported outside America in the African subcontinent and became notorious for its rapid expansion throughout the subcontinent and significant economic losses in maize production in a short span of time^1–3^. In 2018, reports of its presence in the Indian subcontinent were abounded and it was again found to be majorly associated with maize crops across different regions in the country^4–6^. Within a span of 2 years, FAW has established itself throughout most of the regions in India and has been found to attack maize, sorghum, sugarcane, and millets^7–11^. Additionally, FAW invasion has been reported from 19 Asian countries including Myanmar, China, Vietnam, Japan, Korea and in February, 2020, it was detected in Queensland, Australia as well (https://gd.eppo.int/taxon/LAPHFR/distribution). The rapid dispersal of FAW to these widespread regions and varied landscapes is alarming and poses a major threat to the food security efforts of different countries.

Several studies have focussed on genetic analysis to understand the origins of FAW at the newly invaded regions and to ascertain its crop preferences and insecticide resistance potential. *Spodoptera* *frugiperda* is known to exist as two genetically distinct but morphologically identical sister strains, designated as ‘Rice strain’ (R strain) and ‘Corn strain’ (C strain) which differ in their host preferences. R-strain is most consistently found in millet and grass species associated with pasture habitats while the C-strain prefers corn, cotton and sorghum^12,13^. Apart from the variable host distribution, the strains exhibit restricted mating potential and changes in pheromone composition as documented by several studies^14,15^. Owing to similar morphological phenotypes, the two strains of FAW are mainly classified on the basis of polymorphisms in mitochondrial gene *cytochrome c oxidase subunit 1* (COI) and a nuclear gene *triosephosphate isomerase* (*tpi*)^16,17^. The correspondence between the two markers is highly significant for the identification of FAW strains in the Western Hemisphere. However, when the invasive populations from Africa, India and southeast Asia were examined for strain identity by using the two markers, the results were found to be highly discordant. In general, *Tpi* gene was found to accurately predict the host association in invasive populations^3,18,19^. The discordance between the two markers was reflective of the hybrid nature of the invasive populations which was subsequently corroborated with whole-genome studies^20,21^. Barring the detection of two maternal COI lineages and a limited number of *Tpi* haplotypes, genetic variability in the invasive populations was found to be very low. In addition, detection of a unique African *Tpi* ‘rice’ haplotype in the populations from India and south east Asia further strengthened the hypothesis that the invasive populations shared a recent and a common origin^18,19^. Though the notion was challenged by whole mitochondrial sequencing analysis, but there was consensus on highly reduced genetic diversity in invasive populations indicative of a small number of founders^22^.

Most of the studies analyzing the genetic diversity of FAW in India were primarily undertaken in 2018, when FAW was first detected in India and had spread to limited regions within the country^21,22^. The Indian population exhibited high homogeneity with East and South African populations with the majority showing mitochondrial rice strain COI and nuclear corn strain *Tpi* marker^18^. Mitochondrial diversity of the analyzed specimens was found to be very low when compared to the native population^7,21^. Thereafter, the dissemination of FAW in disperse regions, potential subsequent introductions in India and adaptive changes could possibly lead to deviations from the original structure. We were interested in finding whether the Indian FAW populations still conformed to the findings of the previous studies or were they beginning to show digression. This study was designed to capture the diversity of FAW populations in India in the last 2 years, to understand how FAW has adapted to the Indian landscape and to compare the same across the newly expanded range of FAW across the world.

## Results

### Strain identity

**Figure 1:**
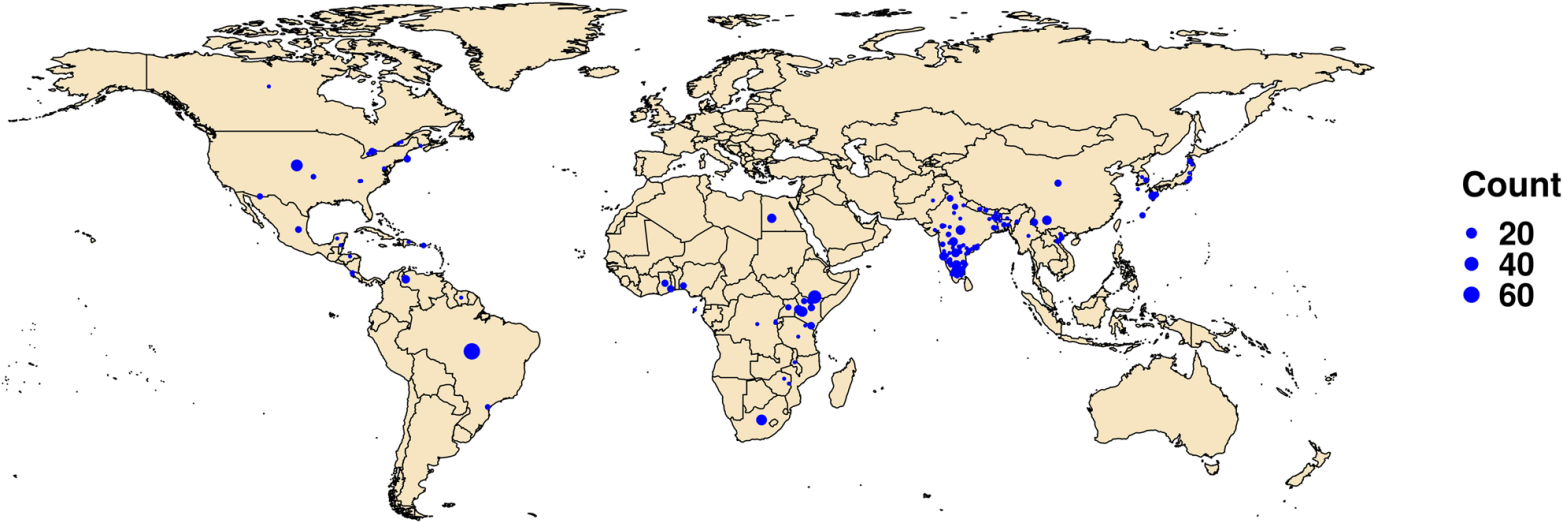
Locations of the FAW samples analysed in this study. The size of the dot is indicative of the number of samples analysed from that location. The map was drawn using R package rworldmap v 1.3.6 and tidyverse v 1.3.0^23,24^.

In India, *S. frugiperda* was found to be a serious pest in maize, sorghum and was occasionally found on sugarcane and cotton in different parts of the country. 92 FAW specimens were collected from 39 different regions in India and Nepal during the year 2018–20 and were subjected to molecular identification at Division of Genomic Resources, ICAR-NBAIR, Bengaluru, India. The generated COI sequences showed 100% similarity with *S. frugiperda* and were deposited in NCBI GenBank database. The sequences and the specimen details were submitted to the BOLD database and DNA barcodes were generated. A list of all COI sequences generated at ICAR-NBAIR is provided in Supplementary File (Table 1a; 1–92). Additional 105 COI sequences which were already deposited in GenBank from different parts of India between 2018 and 2020 were also retrieved and combined with the ICAR-NBAIR dataset to study the diversity of Indian populations. Furthermore, FAW sequences from America (n = 163), Africa (n = 150) and the rest of Asia [China, Japan, Vietnam, Korea, Bangladesh, Pakistan, Myanmar] (n = 76) were collected for comparative sequence analysis and inter-population studies. The approximate locations from which the analyzed specimens were collected are depicted in Fig. 1 and the details of all the retrieved sequences are provided in Supplementary File (Table 1).

We began by investigating the polymorphisms in FAW COI and *Tpi* gene sequences in India, using strain-defining loci and polymorphic sites reported by Nagoshi et al*.*^18^. COIA region or the barcode segment contains polymorphic strain defining locus mCOI602Y, which has been used to distinguish between ‘R’ and ‘C’ sister strains of FAW in the Western Hemisphere. Upon investigation of the mentioned locus, out of 192 COIA sequences from India, 91% of the samples belonged to ‘R’ strain, while the rest belonged to ‘C’ strain. The observed structure is reminiscent of the populations from East Africa and the previously characterized Indian populations from the state of Karnataka^18^. Based on the fact that the specimens were mainly collected from corn crops, it reflects the discordance between the COI marker and the host association of FAW.

**Figure 2:**
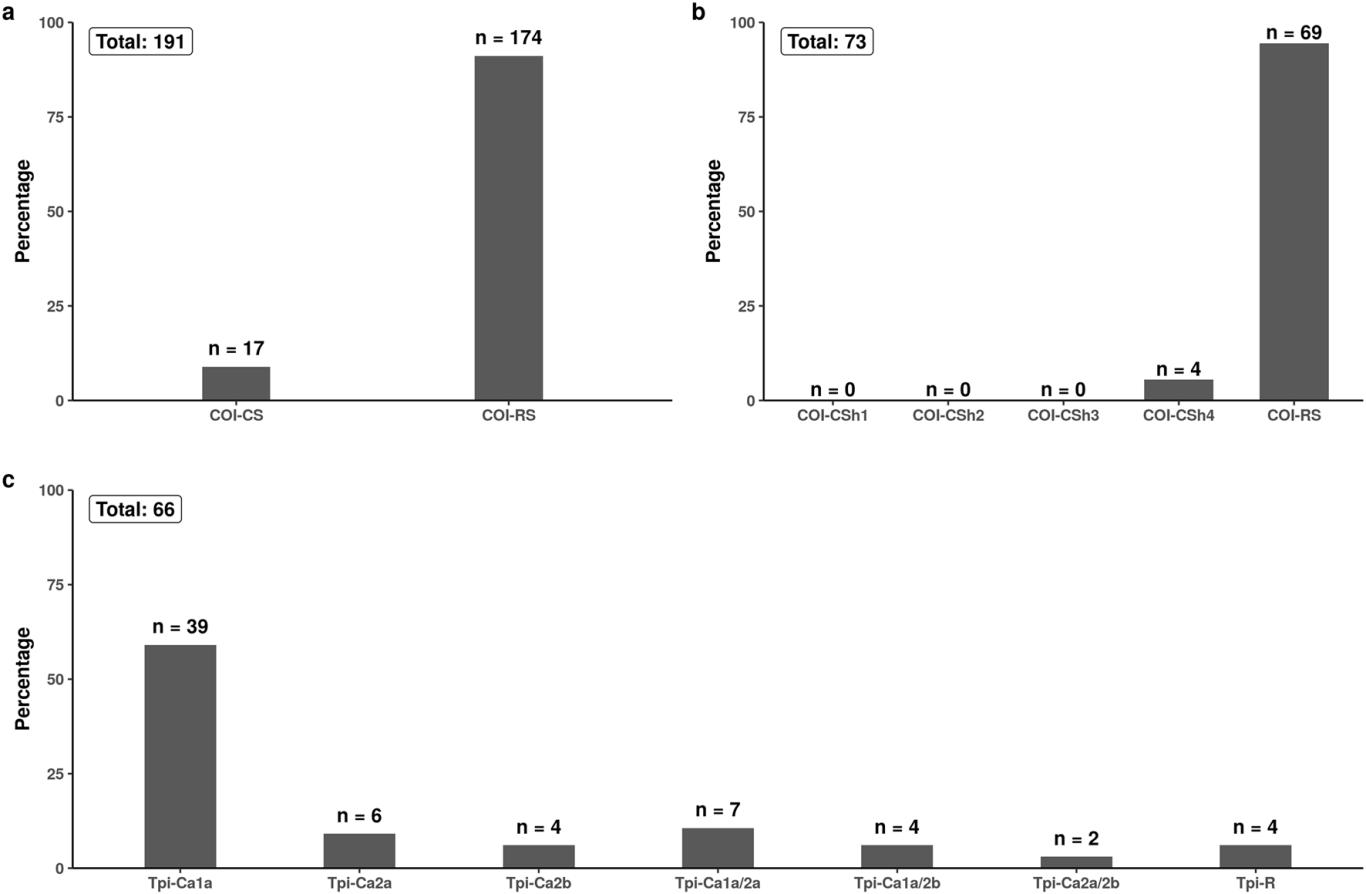
Strain distribution for FAW populations in India based on mitochondrial gene COI and *Tpi* gene markers. (**a**) COIA distribution; (**b**) COIB distribution; (**c**) *Tpi* distribution.

Another strain marker COIB, a segment downstream of the barcode region, was analyzed which contains polymorphic loci mCOI1164D and mCOI1287R giving rise to five haplotypes, four belonging to Corn strain (CSh1-4); and one to the Rice Strain category. The relative distribution of COIB haplotypes, CSh4 and CSh2, has been used as an indicator to study the descent of FAW populations in America. FAW populations collected from Florida and the East coast of America majorly belong to CSh4 haplotype while those from Texas and most other parts of America show a predominance of CSh2 haplotype^25^. COIB haplotyping was done for 72 populations of FAW in India, out of which 68 belonged to ‘R’ strain while the rest demonstrated CSh4 haplotype. We did not find any specimen belonging to other ‘CS’ haplotypes. Overall, the distribution and the composition of both COIA and COIB markers at strain-defining loci were strikingly similar to those of^18^, and do not show much variation in the span of last 2 years (Fig. 2a, b). The list of all the sequences used for COIB analysis are provided in Supplementary File (Table 2).

The polymorphisms at strain-defining loci of the fourth exon and intron segment of *Tpi* gene were studied, which identified four haplotypes in Indian populations (n = 66), i.e. TpiCa1a homozygous (n = 39), TpiCa2a homozygous (n = 6), TpiCa2b homozygous (n = 4) and TpiRa1a homozygous (n = 4). Besides this, thirteen specimens demonstrated the heterozygous corn-strain haplotypes (TpiCa1a/Ca2a; TpiCa1a/Ca2b; TpiCa2a/Ca2b) as observed by their overlapping DNA sequence chromatographs (Fig. 2c). While TpiCa1a is still the predominant haplotype as suggested by Nagoshi et al. (2019), but our data shows higher frequency for TpiCa2a and TpiCa2b haplotypes than that observed earlier^18^. Tpi ‘R’ haplotype was detected from the states of Karnataka and Tripura in India and shared sequence with the unique TpiRa1a haplotype detected from Africa. Akin to the invasive populations from other countries, 90% of the specimens in India exhibited discordant *Tpi* and COI markers for host association prediction^18,19^. 94% of the samples collected from corn crops demonstrated Tpi ‘C’ haplotype. But, the four specimens belonging to Tpi ‘R’ haplotype were also collected from corn fields. So, it is yet to be ascertained whether the TpiRa1a specimens in India show a preference towards rice strain host crops or not. A list of all *Tpi* gene sequences used in this study is provided in Supplementary File (Table 3).

### Polymorphism analysis for FAW populations in India

**Figure 3:**
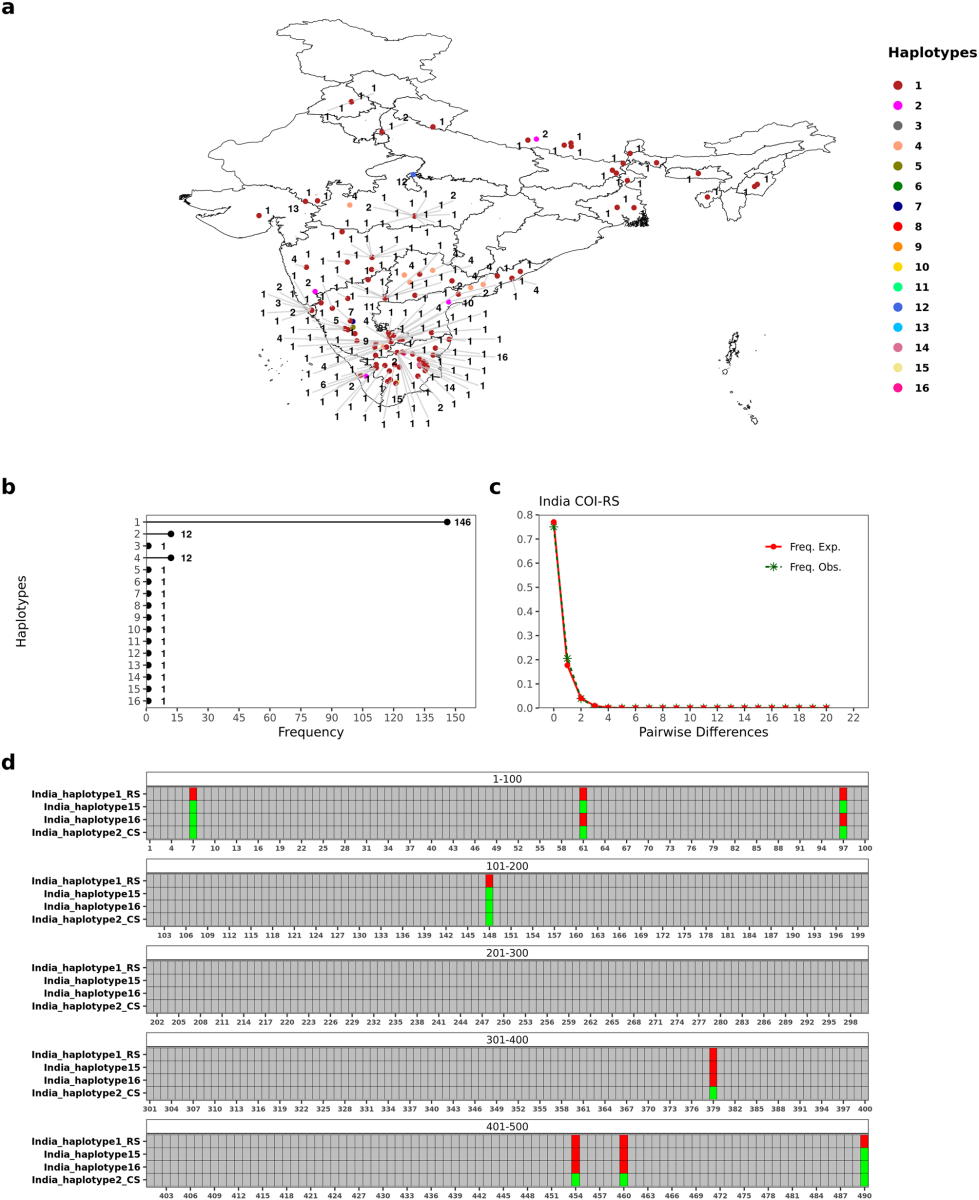
FAW COIA haplotypes from India. (**a**) Distribution of 16 different FAW haplotypes across India. (**b**) Frequency of the different haplotypes found in India. (**c**) Mismatch distribution curve for COIA rice strain haplotypes in India. (**d**) Depiction of polymorphisms in India_haplotype 15 and 16 within 490 bp region of mtCOIA gene with respect to Rice strain (RS) haplotype and a Corn strain (CS) haplotype. India_haplotype 15 and 16 showed characters typical of an inter-strain hybrid haplotype. The figure was drawn using R package tidyverse v 1.3.0 and patchwork v 1.1.1^24,26^.

In order to understand the haplotype diversity of FAW in India, we analyzed 460 bp of COIA barcode region for 183 specimens from India. We were able to find 20 polymorphic sites (21 mutations) in the dataset at a nucleotide diversity of 0.00313. The occurrence of non-synonymous mutations (60%) was higher than the synonymous ones. We also identified 16 different haplotypes from Indian COIA sequences at a haplotype diversity of 0.356, out of which 13 haplotypes belonged to the rice strain while three belonged to the corn strain. As per our knowledge, this study catalogs the highest genetic diversity for FAW reported from India to date. The majority of the analyzed COI sequences belonged to a single rice haplotype (79.7%; India_haplotype 1; n = 146) which was distributed throughout the country. This was followed by the predominant ‘C’ strain haplotype (India_haplotype 2; n = 12) and another ‘R’ strain haplotype from India (India_haplotype 4; n = 12). The remaining haplotypes from India were found as singletons (Fig. 3a, b). We did not find region-specific association of any haplotype in India. Fu’s Fu test and Tajima’s D test statistic were significantly negative, suggesting that the FAW population in India might be undergoing expansion (Table 1). Within ‘R’ strain (n = 169) and ‘C’ strain (n = 14) populations, the nucleotide diversity was higher in ‘C’ strain than ‘R’ strain. Mismatch distribution curve followed a largely unimodal curve for ‘R’ strain populations, representing population expansion within the strain (Fig. 3c). The same was well corroborated with Fu’s Fu and Tajima’s D neutrality tests statistics (Table 2). Surprisingly, we found two gene conversion tracts within India_haplotype 16 and one gene conversion tract in India_haplotype 15, indicative of mitochondrial recombination events between ‘C’ and ‘R’ strain in India (Fig. 3d). Both the haplotypes were identified as ‘C’ strain haplotypes on the basis of mCOI602C locus but demonstrated an inter-strain haplotypic signature. Both the haplotypes were found in close proximity to each other in the Indian state of Tamil Nadu.

**Table 1:** Summary of the genetic diversity of FAW populations analysed on the basis of partial mtCOIA gene from four geographical locations viz. India, America, Africa and Asia-II.

	India	America	Africa	Asia-II	Total
No. of sequences	183	163	150	76	572
No. of sites	459	459	452	460	450
No. of polymorphic sites	20	35	7	9	49
No. of mutations	21	37	7	9	54
No. of haplotypes	16	27	4	5	45
Haplotype diversity	0.356	0.698	0.284	0.383	0.493
Nucleotide diversity	0.00229	0.00772	0.00360	0.00462	0.00562
Fu's Fs statistic	− 9.667	− 8.46	3.864	2.98	− 29.43
Fu and Li's D* test statistic	− 5.04**	− 4.97**	0.241	− 0.099	− 8.98**
Fu and Li's F* test statistic	− 4.58**	− 4.16**	0.457	0.086	− 6.78**
Tajima's D	− 1.93*	− 1.35	0.659	0.41	− 1.86*

(**P < 0.02) (*P < 0.05).

**Table 2:** Comparison between genetic diversity of FAW sister strains in India.

	COIA ‘R’	COIA ‘C’
No. of sequences	169	14
No. of sites	459	460
No. of polymorphic sites	15	5
No. of mutations	15	5
No. of haplotypes	13	3
Nucleotide diversity	0.00065	0.00234
Haplotype diversity	0.250	0.275
Fu's Fs statistic	− 17.677	1.014
Fu and Li's D* test statistic	− 6.75**	− 0.223
Fu and Li's F* test statistic	− 6.08**	− 0.51
Tajima's D	− 2.29**	− 1.09

(**P < 0.02).

### Comparative genetic analysis across the geographical range

We were interested in comparing the diversity of Indian FAW population with that of other geographic regions to study the biogeographic patterning of FAW and to study the relatedness of different sub-populations with each other. For this, we divided the FAW population in four broad groups where population-level studies were feasible. These groups included 1. America (contains population from North and South America); 2. Africa, 3. India and 4. Asia-II (includes populations from Bangladesh, China, Korea, Vietnam, Japan, Myanmar and Pakistan). Along with the previously defined 183 specimens from India, we analyzed 163 COI sequences from America, 150 from Africa and 76 from rest of the Asian countries for polymorphisms. For a comparative study, analysis from all sequences was performed using the same 460 bp region of COI gene which was used for the analysis of Indian populations. In the process, we observed 27, 3, 5 haplotypes, respectively from America, Africa and East-Asia. The predominant ‘R’ and ‘C’ COI haplotypes from America [GenBank Accession: U72977.1, U72974.1] represent the principal haplotypes in all the invaded regions and are presumed to represent the ancestral haplotypes introduced to these regions. Other than the ancestral haplotypes, Africa, India and Asia-II were represented by 2, 14 and 3 haplotypes specific to these regions, respectively. Neutrality test statistics for both African and rest of Asian populations suggested that the populations are still evolving rather neutrally in these places (Table 1).

**Figure 4:**
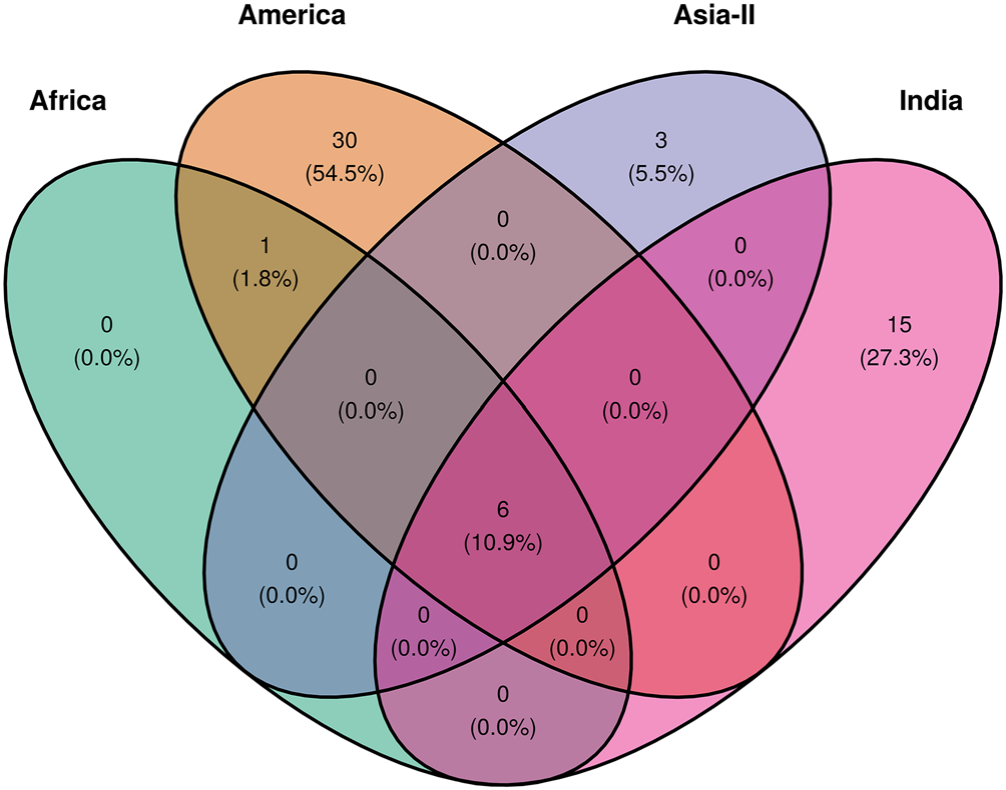
Shared polymorphisms in partial mtCOIA gene for FAW populations across the four geographical groups viz. America, Africa, India and Asia-II. The figure was made with the help of R package tidyverse v 1.3.0 and ggvenn v 0.1.8^24,27^.

We compared the polymorphic sites among the different sub-populations to access the shared polymorphisms across the groups (Fig. 4). We found that African and American populations shared the maximum number of mutations (n = 7), while both India and Asia-II shared six mutations each with the American population. Also among the invading populations, India had the highest number of unshared polymorphisms with the other populations (n = 15), upholding the other statistics regarding population expansion in India.

### Haplotype network and genealogical inferences

**Figure 5:**
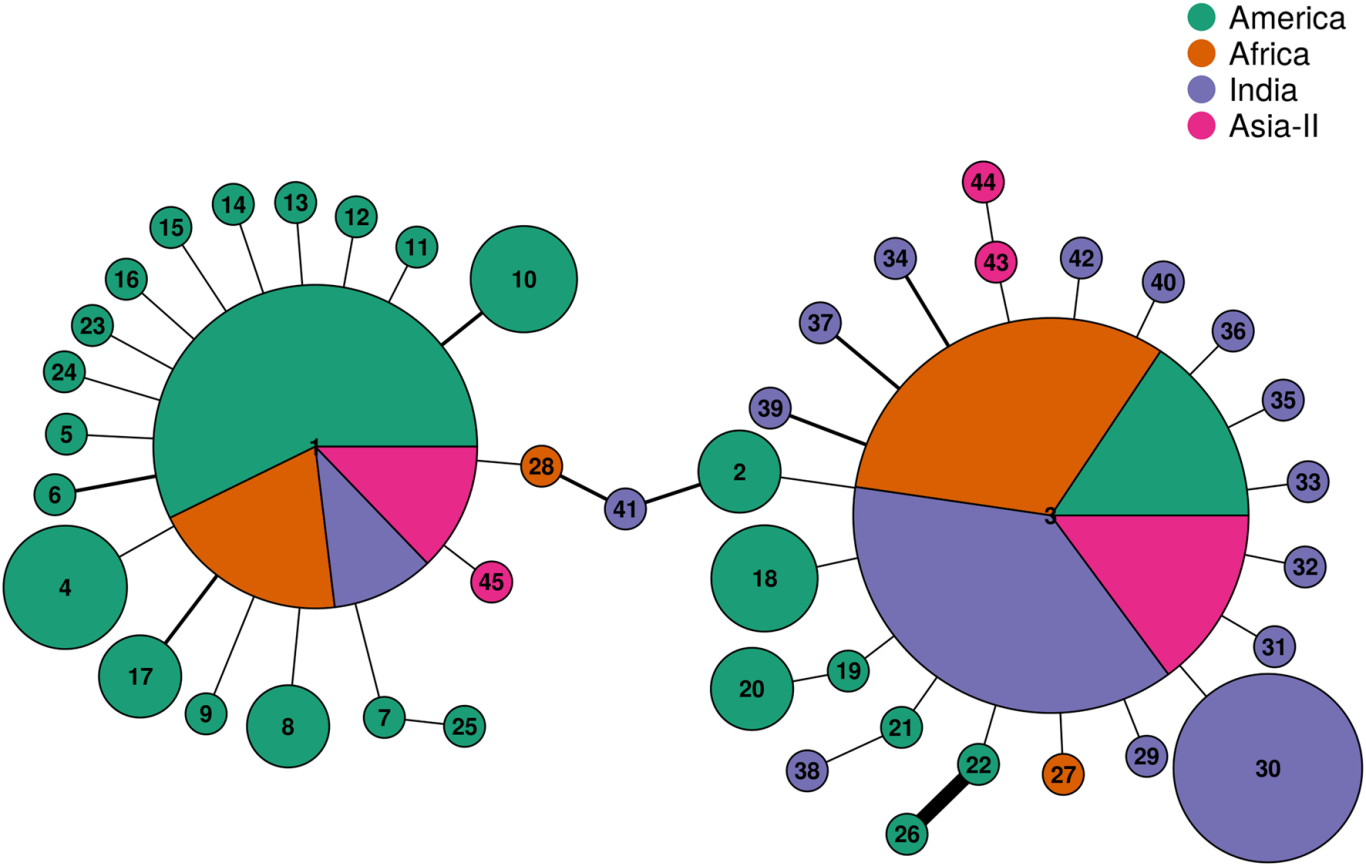
Haplotype network of partial mtCOIA gene sequences of FAW from four geographical groups viz., America, Africa, India and Asia-II. Each pie represents a unique haplotype, the radius of the pie is proportional to the number of sequences belonging to a particular haplotype. The divisions in the pie chart represent the haplotype representation among the four geographical groups. The edge width in the network represent the number of mutation separating the haplotypes from one another, the thinnest edge denotes a single mutation difference.

Haplotypes networks were constructed using haplotypes and their frequencies from populations of America, Africa, India and Asia-II using R-package pegas v0.14. The network constructed for 45 FAW haplotypes across all locations is shown in Fig. 5. The network clearly shows the star-like expansion pattern for the two ancestral rice and corn strain COIA FAW haplotypes. Both the ancestral haplotypes are dominant haplotypes of each of the four geographical regions. However, there are clear differences in the distribution of these haplotypes across the four regions. While the frequency of rice/corn haplotypes in America is 0.41; rice haplotype is the dominant haplotype of the three invaded regions with Africa, India and Asia-II showing frequencies of 0.84, 0.91 and 0.79, respectively. The network suggests the introduction of the two identical maternal lineages at all the invaded regions. Apart from that, there is no evidence to propose multiple introduction events in the invaded regions on the basis of this data. Most of the novel corn haplotypes were found in America whilst most of the novel rice haplotypes were sequenced from India despite much recent invasion of the ancestral rice strain in India. Hap41 from India represent a link between the two ancestral haplotype networks. Hybrid haplotypes of this nature have not been found or reported from any other region before. Notably, gene-conversion tracts were also observed in the same haplotype through polymorphism studies.

### Population structure

Genetic structure between the four geographical groups was analyzsed using AMOVA. We ran seven separate AMOVA analyses, initially comparing the four geographical groups and then the same analysis was performed between different combinations of groups (i.e. America and Africa, America and India, America and Asia-II, Africa and India, Africa and Asia-II, India and Asia-II). The results are shown in Table 3. The results suggest significant genetic differentiation between the four geographical groups (26%) indicating the existence of population structure between regions. In addition to this, significant variation was observed between native American and the invasive populations, with the highest differentiation seen with respect to Indian populations (42.9%). Also, the results predict that both India and Asia-II are genetically more similar to the African population than America.

**Table 3:** Results of AMOVA analysis among the four FAW geographical groups.

Groups	Source	Df	SS	Variance components	Total variance (%)	*P* value
All	Between groups	3	279.485	0.656	26.122	0.001
	Within groups	568	1053.791	1.855	73.878	
	Total	571	1333.276	2.511	100	
America and India	Between groups	1	251.676	1.448	42.957	0.001
	Within groups	344	661.665	1.923	57.043	
	Total	345	913.341	3.372	100	
America and Africa	Between groups	1	152.492	0.961	28.865	0.001
	Within groups	311	736.485	2.368	71.135	
	Total	312	888.978	3.329	100	
Africa and India	Between groups	1	8.597	0.045	3.514	0.005
	Within groups	331	406.273	1.227	96.486	
	Total	332	414.871	1.272	100	
America and Asia-II	Between groups	1	77.065	0.716	20.333	0.001
	Within groups	237	665.186	2.807	79.667	
	Total	238	742.251	3.523	100	
Africa and Asia-II	Between groups	1	1.618	− 0.001	− 0.075	0.282
	Within groups	224	392.126	1.751	100.075	
	Total	225	393.743	1.749	100	
India and Asia-II	Between groups	1	13.258	0.112	8.314	0.001
	Within groups	257	317.306	1.235	91.686	
	Total	258	330.564	1.347	100	

**Figure 6:**
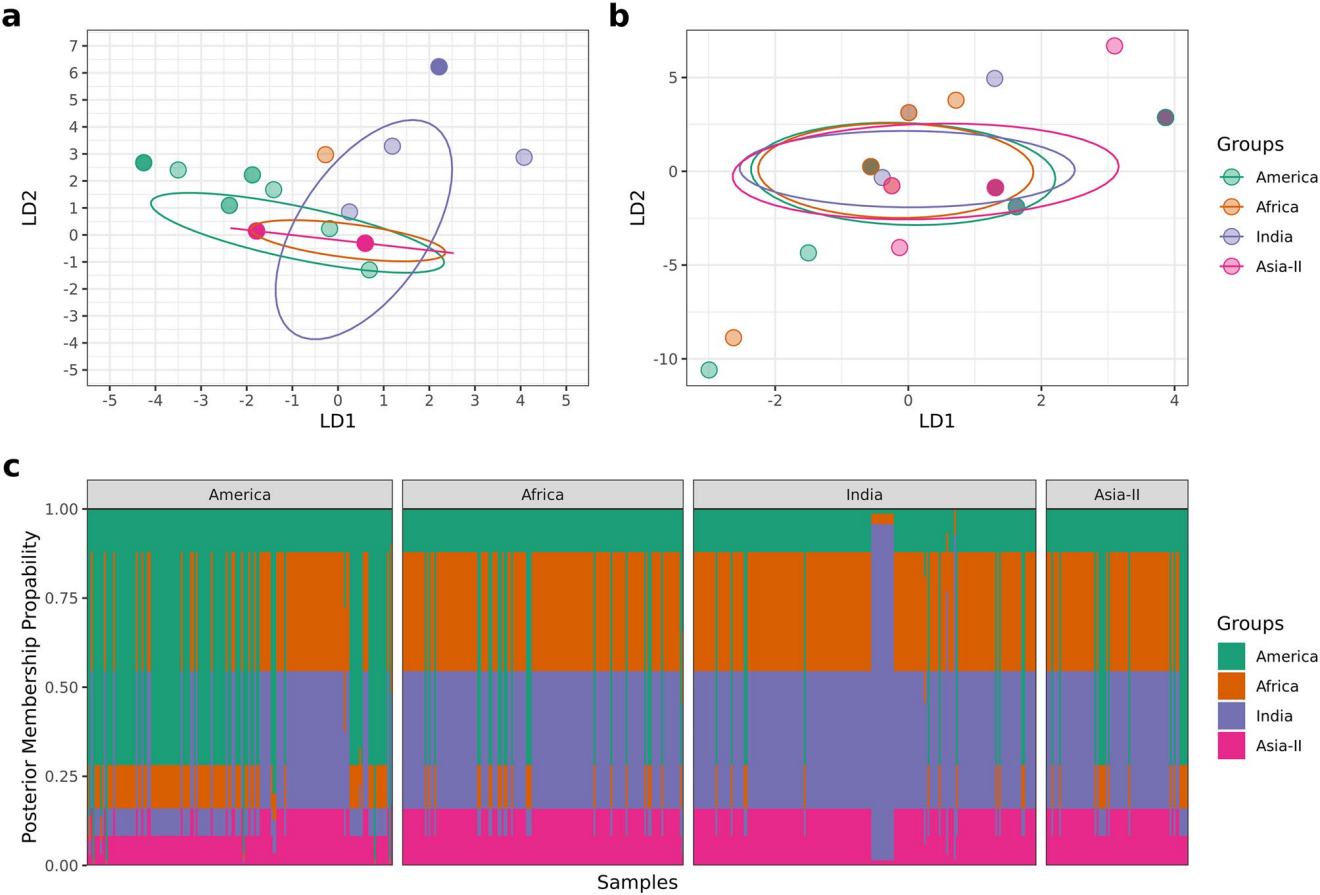
DAPC analysis. (**a**) Clustering of four geographical groups by discriminant analysis of principal components (DAPC) of *S. frugiperda* samples (n = 572). (**b**) DAPC analysis of *S. frugiperda* samples (n = 572) after randomly assigning geographical groups to samples showing no discrimination between clusters. (**c**) Posterior membership probability plot shows posterior probability assignment of samples to their respective geographical groups.

Similar results were corroborated with DAPC analysis and the resultant membership table which indicated two clear discriminant clusters between America and India (Fig. 6).

## Discussion

An ideal design for any population genomics study would involve diligent tracing of the evolutionary course of a population over geography or time. *Spodoptera frugiperda* provides an excellent model system in this regard since it is believed to have expanded its distribution range very recently and has been well documented and characterized for its diversity in its native as well as the extended range. Most of the studies analysing the diversity of Indian FAW populations were performed around 2 years ago from a few locations where it was first detected in India^7,18,21,22^. Thus we thought it was imperative to understand the diversity of the Indian FAW population over the course of the last 2 years after the expansive migration of FAW across India.

During our preliminary investigation of strain defining loci from COI and *Tpi* genes, we did not find much variation between our dataset and the previous study from India by Nagoshi et al*.*^18^. The structure and composition of the FAW strains for COI locus was well preserved over this period. We found a disagreement of nearly 90% between COI and *Tpi* gene markers for the prediction of host association as has been ubiquitously reported for the populations from most of the newly invaded regions across the world^3,18,19^. *Tpi* gene loci provided a better estimate for host preference in the specimens investigated in our study.

When polymorphisms in mitochondrial COIA gene were studied in Indian populations, we found several novel haplotypes predominantly belonging to the COIA ‘R’ strain, which were unique to India. Several population genetic test statistics suggested population expansion in India while indicating that the populations in Africa and other East Asian countries were still evolving neutrally. We tried to investigate the reasons behind the same. First, we analyzed the distribution of different haplotypes in India to understand the impact of regional influences in shaping the genetic diversity in India. India is considered as one of the most biodiverse countries of the world and contains four major biodiversity hotspots in a dense cluster^28,29^. It is divided into 15 different agro-climatic zones on the basis of soil type, climate, water availability etc. (http://mowr.gov.in/agro-climatic-zones), which is partly responsible for the observed diversity in India. Previous studies on the intra-species diversity of certain insect pests in India have suggested geographical sub-structuring within different regions of India^30–32^. We looked at the distribution of the different haplotypes in India but most of the novel haplotypes in India at present are too infrequent and dispersed to attribute them to specific ecozones of India. Moreover, looking at the rampant distribution of the predominant rice and corn haplotypes throughout India, the migration of FAW within India does not anyhow seem restricted. However, future studies would help to shed more light on regional associations or microcosms of different haplotypes in India and their adaptive advantage over other haplotypes in the Indian landscape.

As an alternate hypothesis, we tried to investigate whether multiple introductions of FAW could contribute to the higher diversity seen in India. In this regard, shared polymorphism analysis or network studies from partial COIA sequences did not provide any evidence for multiple introductions to India. Most of the novel haplotypes seen in India belonged to the COIA ‘R’ strain category and were not found in America or any other part of the world. In fact, India, Africa and other regions of Asia shared common ancestral haplotypes and similar polymorphisms with each other, signifying a common source for all these populations. However, a recent study involving whole-genome sequencing of FAW populations from New World and invaded regions has proposed multiple origins for FAW in the invaded countries^22^. On that basis, we think it would be prudent to analyze the diversity in Indian populations with multiple gene loci to conclusively trace the geographic origins or monitor the outflows from India. However, based on our analysis from a large dataset of FAW COI sequences, we found that the mtCOI gene was useful in sub-structuring the populations from different geographical groups but did not indicate introduction of multiple COI haplotypes to India.

Conversely, on a different note, it is important to state that within our primary data generated from 92 FAW specimens collected from 39 different regions of India, we did not observe similar diversity levels as those found in other FAW sequences reported from India. We could only find the two ancestral COIA ‘rice’ and ‘corn’ haplotypes which are reported from invasive populations of Africa and East Asia. The observed haplotype diversity was much in line with that found in Africa (Supplementary File, Table 4). So, it is possible that with more sampling from India, the reported diversity levels get normalized and come more in accordance with those found at other newly invaded countries.

Two haplotypes found in Indian populations were particularly interesting and represented a hybrid between the rice and corn strains. The haplotypes appear to originate from gene conversion events by recombination between the strains. Though, the polymorphisms could be attributed to mutation events, but it is unlikely to observe as many mutations as shown by these haplotypes specifically in the parsimony informative sites for FAW strain identification. Recombination in animal mitochondrial DNA, though sporadic, has been observed in several natural populations and inter-specific crosses^33–35^. Higher levels of divergence between the haplotypes or species aid in the identification of these events, which might otherwise remain unnoticed. In the likely scenario of a gene recombination event, the presence of these haplotypes suggests that inter-strain matings between the COIA ‘R’ and ‘C’ strains are occurring in the field conditions. But it is important to understand that COIA’R’ strain found in India or other recently invaded countries does not represent the true ancestral ‘R’ strain. Multiple studies have suggested it to be a hybrid strain as marked by the discordance between *Tpi* and COIA markers and by other genome sequencing studies^18,20,21^. Thus, the behavior of this population is not well characterized and the mating barriers between this population and the ancestral strains are also not yet known. Nevertheless, it would be fascinating to study the behavior of the inter-strain hybrid COIA haplotypes found in India and future studies might help us in assessing any potential benefits of these mating events for the evolution of FAW in India.

This is amongst the first studies to demonstrate the genetic diversity of FAW across its entire geographical range after its recent invasions to other parts of the world. The data suggests that India might be emerging as the new hotspot for the expansion of FAW particularly for the COIA rice haplotypes. Inter-strain recombination has also been noticed in India, which opens up a possible new evolutionary trajectory. As FAW has started expanding its host range in India, this study would prove helpful to ascertain the association between host range and the novel FAW haplotypes detected in India. Future studies would also focus on assessing the control efficacy of different insecticides and biocontrol agents on the different FAW haplotypes in India. Lastly, this study would serve as a good reference to track the subsequent evolution and spread of FAW in India as well as the rest of the demographic range.

## Methods

### Sample collection, DNA extraction and PCR amplification

92 FAW specimens were collected from 39 different regions in India and Nepal during the years 2018–2020. The larvae from each collection were stored in absolute ethanol at − 80 °C until DNA extraction. Genomic DNA was isolated from single larva by using QIAGEN DNeasy blood and tissue kit, Germany, following the manufacturer’s protocols. The remaining larvae were kept as voucher specimens at − 80 °C. DNA thus obtained was subjected to PCR amplification using a Bio-Rad C1000 Thermal Cycler. Each PCR reaction of 50 μL consisted of 5 μL 10X DreamTaq master mix, 2 μL of 10 mM dNTP mix, 1 μL (20 pmol/μL) each of gene-specific forward and reverse primer (COIA: LCO1490 and HCO2198, 658 bp^36^; COIB: 891F and 1472R; *Tpi* gene: 412F and 1140R^18^), 0.5 μL DreamTaq DNA polymerase (5 U/μL), 5 μL DNA (50 ng/μL), and 35 μL sterile water. Thermo-cycling parameters used for the study consisted of an initial denaturation of 94 °C for 5 min, followed by 30 cycles of denaturation at 94 °C for 1 min, annealing at specific temperatures for 1 min, extension at 72 °C for 1 min. The amplified products were analyzed on 1.5% agarose gel electrophoresis. Each specimen PCR sample was bi-directionally sequenced and checked for homology, insertions and deletions, stop codons, and frame shifts by using NCBI-BLAST and ORF finder. The COIA generated sequences showed 100% similarity as *S. frugiperda* and were then deposited in NCBI GenBank database and accession number was retrieved for all the populations. Further the sequences and the specimen details were submitted to the BOLD database and DNA barcodes were generated.

### DNA polymorphism analysis

105 additional mitochondrial COIA sequences deposited from India during the year 2018–2020 were retrieved from GenBank. These sequences were aligned along with the sequences generated at  ICAR-NBAIR with MEGA-X *ver*. 10.1.7 using ClustalW^37^. The aligned sequences were trimmed to 460 bp length since this region was commonly present in 183 sequences from India. The rest of the COIA sequences which did not contain the selected 460 bp region were removed from further analysis. The final list of COI sequences used for the polymorphism analysis is provided in Supplementary File (Table 1a). Additionally, 163 COI sequences deposited from America, 150 from Africa and 76 from rest of the Asian countries were retrieved from GenBank and were trimmed to the same length as the Indian sequences and used for polymorphism studies. The dataset was divided into four broad categories as (1) America, (2) Africa, (3) India, (4) Asia-II based on the geographical distribution. These groups were analyzed for descriptive statistics such as nucleotide diversity, number of haplotypes (H), haplotype diversity (Hd), genetic neutrality tests and mismatch distribution analysis using DnaSP *ver* 6.12.03^38^.

### Network plot

Haplotype network plot was constructed using the R package pegas v0.14^39,40^. The package uses a parsimony network described by^41^ for establishing relationship among haplotypes. Each pie represents a unique haplotype; the radius of the pie is proportional to the number of sequences belonging to a particular haplotype. We transformed the number of sequences with log2 + 1, to enable optimal visualization of haplotypes with a single sequence.

### AMOVA

Population structure was studied from the alignment file using AMOVA^42^. Analysis of molecular variance (AMOVA) was performed using the R package poppr v2.8.6^43^. The hierarchy for this analysis was based on four predetermined geographical regions America, Africa, India and Asia-II. The percentage of observed variance within and between groups was calculated initially among all the regions and then the same analysis was performed between different combinations of two groups (America and Africa, America and India, America and Asia-II, Africa and India, Africa and Asia-II, India and Asia-II). The significance of observed variance between groups was tested by a randomization test with 1000 permutations. This test was performed using the accompanying R package ade4 v1.7.15 and the function randtest^44^.

### DAPC

A second method discriminant analysis of principal components, was used to further study the population structure. This analysis was performed using the R package adegnet v2.1.3^45,46^. This method was applied to the aligned sequence file to find the extent of discrimination between different predefined groups. The balance between discrimination power and overfitting was maintained by selecting the optimal number of principal components for analysis using the a-score optimization test from the function optim.a.score. We have retained eight principal components as the optimum number based on the a-score optimization test. As there are four geographical groups, we retained three discriminant functions. Predefined groups were then randomized and the analysis was repeated with the new randomized data to confirm that discrimination between clusters does not occur by chance. DAPC analysis was also used to study the level of admixture in the predefined groups. Samples with less than 50% posterior membership probability were considered admixed while the samples with more than 50% posterior membership probability were allotted with their respective group.

## Supplementary Information

Supplementary Information.
