# Supplementary material for: Population structure and genetic diversity of invasive Fall Armyworm after 2 years of introduction in India
Source: Sci Rep. 2021 Apr 8;11:7760. doi: 10.1038/s41598-021-87414-5 (PMC8032663; doi:10.1038/s41598-021-87414-5)
Supplement: Supplementary file 1 — Supplementary Information. [file 41598_2021_87414_MOESM1_ESM.docx]

Supplementary File

Table 1: Details of *Spodoptera frugiperda* COIA gene sequences used in the present study.

A) COIA gene sequences from India

| S.No. | GenBank Accession | Location | Year  Submitted | Year Collected | Used for DNAsp | Used for Strain Identification |
| --- | --- | --- | --- | --- | --- | --- |
| 1. | MN264674.1 | Tirupati | 2019 |  | ✓ | ✓ |
| 2. | MT872401.1 | Gouribidanur | 2020 |  | ✓ | ✓ |
| 3. | MN117908.1 | Malada | 2019 | 2019 | ✓ | ✓ |
| 4. | MK285364.1 | Pune | 2019 |  | ✓ | ✓ |
| 5. | MH881533.1 | Hassan | 2019 | 2018 | ✓ | ✓ |
| 6. | MH881532.1 | Bellur | 2019 |  | ✓ | ✓ |
| 7. | MH881531.1 | Shimoga | 2019 |  | ✓ | ✓ |
| 8. | MH881530.1 | Rajendranagar | 2019 |  | ✓ | ✓ |
| 9. | MH881529.1 | Siddipet | 2019 |  | ✓ | ✓ |
| 10. | MH881528.1 | Nagarkurnool | 2019 |  | ✓ | ✓ |
| 11. | MH822835.1 | Khammam | 2019 | 2018 | ✓ | ✓ |
| 12. | MH822834.1 | Nelivada | 2019 | 2018 | ✓ | ✓ |
| 13. | MH822833.1 | Pedabathevalasa | 2019 | 2018 | ✓ | ✓ |
| 14. | MH822832.1 | Chinthapalle | 2019 | 2018 | ✓ | ✓ |
| 15. | MN126574.1 | Anakapalle | 2019 |  | ✓ | ✓ |
| 16. | MH822830.1 | Dharwad | 2019 | 2018 | ✓ | ✓ |
| 17. | MH899611.1 | Vijayawada | 2018 |  | ✓ | ✓ |
| 18. | MH899610.1 | Tirupati | 2018 |  | ✓ | ✓ |
| 19. | MH899609.1 | Pune | 2018 |  | ✓ | ✓ |
| 20. | MT906152.1 | Darjeeling | 2020 |  | ✓ | ✓ |
| 21 | MT886717.1 | Delhi | 2020 |  | ✓ | ✓ |
| 22. | MT906149.1 | Delhi | 2020 |  | ✓ | ✓ |
| 23. | MT901173.1 | Kerala | 2020 |  | ✓ | ✓ |
| 24. | MN218588.1 | West Bengal | 2019 |  | ✓ | ✓ |
| 25. | MN218589.1 | Cooch Behar | 2019 | 2019 | ✓ | ✓ |
| 26. | MN233305.1 | Cooch Behar | 2019 | 2019 | ✓ | ✓ |
| 27. | MH704433.1 | Chikkabalapur | 2019 | 2018 | ✓ | ✓ |
| 28. | MK559414.1 | Mandya | 2019 | 2019 | ✓ | ✓ |
| 29. | MK327538.1 |  | 2019 |  | ✓ | ✓ |
| 30. | MK318531.1 |  | 2019 |  | ✓ | ✓ |
| 31. | MK303391.1 | Tapi | 2019 |  | ✓ | ✓ |
| 32. | MK279399.1 | Tapi | 2019 |  | ✓ | ✓ |
| 33. | MK079565.1 | Chikkabalapur | 2019 |  | ✓ | ✓ |
| 34. | MT664985.1 | Bishnupur | 2020 | 2019 | ✓ | ✓ |
| 35. | MT872404.1 | Bengaluru | 2020 |  | ✓ | ✓ |
| 36. | MT872402.1 | Bengaluru | 2020 |  | ✓ | ✓ |
| 37. | MT872403.1 | Bengaluru | 2020 |  | ✓ | ✓ |
| 38. | MT872400.1 | Bengaluru | 2020 |  | ✓ | ✓ |
| 39. | MT872399.1 | Bengaluru | 2020 |  | ✓ | ✓ |
| 40. | MT901175.1 | Katihar | 2020 |  | ✓ | ✓ |
| 41. | MT901174.1 | Katihar | 2020 |  | ✓ | ✓ |
| 42. | MT906150.1 | Kishanganj | 2020 |  | ✓ | ✓ |
| 43. | MT906151.1 | Darjeeling | 2020 |  | ✓ | ✓ |
| 44. | MT901176 | Tripura | 2020 |  | ✓ | ✓ |
| 45. | MT936877.1 | Bagalkot | 2020 |  | ✓ | ✓ |
| 46. | MT936878.1 | Chikkabalapur | 2020 |  | ✓ | ✓ |
| 47. | MT936879.1 | Doddabalapur | 2020 |  | ✓ | ✓ |
| 48. | MN584897 | Maharashtra | 2020 |  | ✓ | ✓ |
| 49. | MN584898 | Nepal | 2020 |  | ✓ | ✓ |
| 50. | MN584899 | Khumaltar | 2020 |  | ✓ | ✓ |
| 51. | MN584900 | Nawalpur | 2020 |  | ✓ | ✓ |
| 52. | MK041922.1 | Kolar | 2019 |  | ✓ | ✓ |
| 53. | MW193890 | West Bengal | 2020 |  | ✓ | ✓ |
| 54. | MW561292 | Pennagaram | 2021 | 2020 | ✓ | ✓ |
| 55. | MW561293 | Pennagaram | 2021 | 2020 | ✓ | ✓ |
| 56. | MW561294 | Pennagaram | 2021 | 2020 | ✓ | ✓ |
| 57. | MW561295 | Pennagaram | 2021 | 2020 | ✓ | ✓ |
| 58. | MW561296 | Pennagaram | 2021 | 2020 | ✓ | ✓ |
| 59. | MW561297 | Pennagaram | 2021 | 2020 | ✓ | ✓ |
| 60. | MW561298 | Pennagaram | 2021 | 2020 | ✓ | ✓ |
| 61. | MW561299 | Pennagaram | 2021 | 2020 | ✓ | ✓ |
| 62. | MW561300 | Pennagaram | 2021 | 2020 | ✓ | ✓ |
| 63. | MW561301 | Pennagaram | 2021 | 2020 | ✓ | ✓ |
| 64. | MW561302 | Raichur | 2021 | 2020 | ✓ | ✓ |
| 65. | MW561303 | Raichur | 2021 | 2020 | ✓ | ✓ |
| 66. | MW561304 | Raichur | 2021 | 2020 | ✓ | ✓ |
| 67. | MW561305 | Raichur | 2021 | 2020 | ✓ | ✓ |
| 68. | MW561306 | Raichur | 2021 | 2020 | ✓ | ✓ |
| 69. | MW561307 | Raichur | 2021 | 2020 | ✓ | ✓ |
| 70. | MW561308 | Raichur | 2021 | 2020 | ✓ | ✓ |
| 71. | MW561309 | Raichur | 2021 | 2020 | ✓ | ✓ |
| 72. | MW561310 | Raichur | 2021 | 2020 | ✓ | ✓ |
| 73. | MW561311 | Raichur | 2021 | 2020 | ✓ | ✓ |
| 74. | MW561312 | Parbhani | 2021 | 2020 | ✓ | ✓ |
| 75. | MW561313 | Parbhani | 2021 | 2020 | ✓ | ✓ |
| 76. | MW561314 | Parbhani | 2021 | 2020 | ✓ | ✓ |
| 77. | MW561315 | Parbhani | 2021 | 2020 | ✓ | ✓ |
| 78. | MW561316 | Parbhani | 2021 | 2020 | ✓ | ✓ |
| 79. | MW561317 | Parbhani | 2021 | 2020 | ✓ | ✓ |
| 80. | MW561318 | Parbhani | 2021 | 2020 | ✓ | ✓ |
| 81. | MW561319 | Parbhani | 2021 | 2020 | ✓ | ✓ |
| 82. | MW561320 | Parbhani | 2021 | 2020 | ✓ | ✓ |
| 83. | MW561321 | Parbhani | 2021 | 2020 | ✓ | ✓ |
| 84. | MW561322 | Chickballapur | 2021 |  | ✓ | ✓ |
| 85. | MW561323 | Kolar | 2021 |  | ✓ | ✓ |
| 86. | MW561324 | Kolar | 2021 |  | ✓ | ✓ |
| 87. | MW561325 | Doddaballapur | 2021 |  | ✓ | ✓ |
| 88. | MW561326 | Kanakapura | 2021 |  | ✓ | ✓ |
| 89. | MW624694 | Ludhiana | 2020 | 2020 | ✓ | ✓ |
| 90. | MW624695 | Ludhiana | 2020 | 2020 | ✓ | ✓ |
| 91. | MW624696 | Ludhiana | 2020 | 2020 | ✓ | ✓ |
| 92. | MW624697 | Ludhiana | 2020 | 2020 | ✓ | ✓ |
| 93. | MN640599.1 | Meghalaya | 2020 |  | ✓ | ✓ |
| 94. | MN640598.1 | Meghalaya | 2020 |  | ✓ | ✓ |
| 95. | MK908223.1 | Anakapalle | 2019 |  | X | ✓ |
| 96. | MK368810.1 | Goa | 2019 |  | ✓ | ✓ |
| 97. | MH819361.1 | East Godavari | 2019 |  | ✓ | ✓ |
| 98. | MH819360.1 | Ujjain | 2019 |  | ✓ | ✓ |
| 99. | MH819359.1 | Pune | 2019 |  | ✓ | ✓ |
| 100. | MH819358.1 | Anakapalle | 2019 |  | ✓ | ✓ |
| 101. | MH819357.1 | Medak | 2019 |  | ✓ | ✓ |
| 102. | MH819356.1 | West Godavari | 2019 |  | ✓ | ✓ |
| 103. | MH819355.1 | Hyderabad | 2019 |  | ✓ | ✓ |
| 104. | MH819354.1 | Jammikunta | 2019 |  | ✓ | ✓ |
| 105. | MH819353.1 | Hassan | 2019 |  | ✓ | ✓ |
| 106. | MH819352.1 | Bengaluru | 2019 |  | ✓ | ✓ |
| 107. | MH753334.1 | Bengaluru | 2018 | 2018 | ✓ | ✓ |
| 108. | MH753333.1 | Haniyuru | 2018 | 2018 | ✓ | ✓ |
| 109. | MH753332.1 | Hanchipura | 2018 | 2018 | ✓ | ✓ |
| 110. | MH753331.1 | Chennagiri | 2018 | 2018 | ✓ | ✓ |
| 111. | MH753330.1 | Badravathi | 2018 | 2018 | ✓ | ✓ |
| 112. | MH753329.1 | Belagavi | 2018 | 2018 | ✓ | ✓ |
| 113. | MH753328.1 | Coimbatore | 2018 | 2018 | ✓ | ✓ |
| 114. | MH753327.1 | Kaduru | 2018 | 2018 | ✓ | ✓ |
| 115. | MH753326.1 | Shimoga | 2018 | 2018 | ✓ | ✓ |
| 116. | MH753325.1 | Honalli | 2018 | 2018 | ✓ | ✓ |
| 117. | MH753324.1 | Chamaraja nagar | 2018 | 2018 | ✓ | ✓ |
| 118. | MH753323.1 | Davanagare | 2018 | 2018 | ✓ | ✓ |
| 119. | MH639007.1 | Bengaluru | 2018 | 2018 | ✓ | ✓ |
| 120. | MH639008.1 | Bengaluru | 2018 | 2018 | ✓ | ✓ |
| 121. | MH639006.1 | Bengaluru | 2018 | 2018 | ✓ | ✓ |
| 122. | MH639005.1 | Bengaluru | 2018 | 2018 | ✓ | ✓ |
| 123. | MH639004.1 | Bengaluru | 2018 | 2018 | ✓ | ✓ |
| 124. | MT412119.1 | Pallakad | 2020 | 2019 | ✓ | ✓ |
| 125. | MT380064.1 | Pallakad | 2020 | 2019 | X | ✓ |
| 126. | MT219815.1 | Pallakad | 2020 | 2019 | X | ✓ |
| 127. | MT189271.1 |  | 2020 |  | X | ✓ |
| 128. | MT189270.1 |  | 2020 |  | X | ✓ |
| 129. | MT189269.1 |  | 2020 |  | X | ✓ |
| 130. | MT189268.1 |  | 2020 |  | X | ✓ |
| 131. | MT189267.1 |  | 2020 |  | X | ✓ |
| 132. | MT189266.1 |  | 2020 |  | X | ✓ |
| 133. | MT185360.1 |  | 2020 |  | X | ✓ |
| 134. | MT185359.1 |  | 2020 |  | X | ✓ |
| 135. | MT074051.1 | Kalyani | 2020 | 2019 | X | ✓ |
| 136. | MN888504.1 | Jhansi | 2020 | 2019 | ✓ | ✓ |
| 137. | MK608013.1 | Punawara | 2019 | 2019 | ✓ | ✓ |
| 138. | MK295625.1 | Kolhapur | 2019 | 2019 | ✓ | ✓ |
| 139. | MK106259.1 | Harihara | 2018 | 2019 | ✓ | ✓ |
| 140. | MK106258.1 | Shimoga | 2018 | 2019 | ✓ | ✓ |
| 141. | MK591010.1 | Punawara | 2019 | 2019 | ✓ | ✓ |
| 142. | MT605970.1 | Modakurichi | 2020 |  | ✓ | ✓ |
| 143. | MT605971.1 | Kagithapuram | 2020 |  | ✓ | ✓ |
| 144. | MN541574.1 | Delhi | 2020 |  | ✓ | ✓ |
| 145. | MT221443.1 | Palakkad | 2020 |  | X | ✓ |
| 146. | MT216515.1 | Perambulur | 2020 | 2019 | ✓ | ✓ |
| 147. | MT216499.1 | Tiruchirapalli | 2020 | 2019 | ✓ | ✓ |
| 148. | MT215104.1 | Paithanth | 2020 | 2019 | ✓ | ✓ |
| 149. | MT215033.1 | Chelampatti | 2020 |  | ✓ | ✓ |
| 150. | MT215034.1 | Dharmapuri | 2020 |  | ✓ | ✓ |
| 151. | MT215032.1 | Paithanth | 2020 | 2019 | ✓ | ✓ |
| 152. | MT215030.1 | Tiruppur | 2020 | 2019 | ✓ | ✓ |
| 153. | MT215031.1 | Dharmapuri | 2020 | 2019 | ✓ | ✓ |
| 154. | MT215029.1 | Pudukottai | 2020 |  | ✓ | X |
| 155. | MT215019.1 | Pollachi | 2020 | 2019 | X | X |
| 156. | MT199124.1 | Kodangipatti | 2020 | 2019 | ✓ | ✓ |
| 157. | MT199123.1 | Ariyalur | 2020 | 2019 | ✓ | X |
| 158. | MT199119.1 | Pudur | 2020 |  | ✓ | X |
| 159. | MT199122.1 | Ariyalur | 2020 | 2019 | ✓ | ✓ |
| 160. | MT199118.1 | Madurai | 2020 |  | ✓ | ✓ |
| 161. | MT199117.1 | Edayarpalayam | 2020 |  | ✓ | ✓ |
| 162. | MT199121.1 | Perumugai | 2020 | 2019 | ✓ | ✓ |
| 163. | MT199113.1 | Dharmapuri | 2020 |  | ✓ | ✓ |
| 164. | MT199116.1 | Vannathiparai | 2020 | 2019 | ✓ | X |
| 165. | MT199120.1 | Pudukadu | 2020 |  | ✓ | X |
| 166. | MT199112.1 | Palakkad | 2020 |  | ✓ | ✓ |
| 167. | MT199115.1 | Perambulur | 2020 | 2019 | ✓ | ✓ |
| 168. | MT199114.1 | Thanjavur | 2020 | 2019 | ✓ | ✓ |
| 169. | MT199108.1 | Virudhunagar | 2020 | 2019 | ✓ | ✓ |
| 170. | MT199110.1 | Naduvappacheri | 2020 |  | ✓ | ✓ |
| 171. | MT199109.1 | Mattangal | 2020 |  | ✓ | ✓ |
| 172. | MT199107.1 | Sivaganga | 2020 |  | ✓ | ✓ |
| 173. | MN117927.1 |  | 2020 | 2019 | ✓ | ✓ |
| 174. | MN486495.1 |  | 2020 |  | ✓ | ✓ |
| 175. | MN486494.1 |  | 2020 |  | ✓ | ✓ |
| 176. | MN486493.1 |  | 2020 |  | ✓ | ✓ |
| 177. | MN486492.1 |  | 2020 |  | ✓ | ✓ |
| 178. | MN486491.1 |  | 2020 | 2019 | ✓ | ✓ |
| 179. | MK633906.1 | Banswara | 2020 | 2019 | ✓ | ✓ |
| 180. | MK034861.1 | Anand | 2019 | 2018 | ✓ | ✓ |
| 181. | MN630563.1 | Pantnagar | 2020 |  | ✓ | ✓ |
| 182. | MT644266.1 | Pune | 2020 |  | ✓ | ✓ |
| 183. | MN615884.1 | Manipur | 2020 |  | ✓ | ✓ |
| 184. | MN187007.1 |  | 2019 | 2019 | ✓ | ✓ |
| 185. | MN218588.1 | West Bengal | 2019 | 2019 | ✓ | ✓ |
| 186. | MK105750.1 | Harihara | 2019 | 2018 | ✓ | ✓ |
| 187. | MK105749.1 | Shimoga | 2019 | 2018 | ✓ | ✓ |
| 188. | MN011579.1 | Nepal | 2020 |  | ✓ | ✓ |
| 189. | MT103345.1 | Bhakundebesi | 2020 |  | ✓ | ✓ |
| 190. | MT791636.1 | Goa | 2020 |  | ✓ | ✓ |
| 191. | MT791635.1 | Goa | 2020 |  | ✓ | ✓ |
| 192. | MT791633.1 | Goa | 2020 |  | ✓ | ✓ |
| 193. | MT791632.1 | Goa | 2020 |  | ✓ | ✓ |
| 194. | MT791631.1 | Goa | 2020 |  | ✓ | ✓ |
| 195. | MT791630.1 | Goa | 2020 |  | ✓ | ✓ |
| 196. | MT791629.1 | Goa | 2020 |  | ✓ | ✓ |
| 197. | MT791628.1 | Goa | 2020 |  | ✓ | ✓ |

B) COIA gene sequences from America

| S.No. | GenBank Accession | Location | Year  Submitted |
| --- | --- | --- | --- |
| 1. | KX281221.1 |  | 2017 |
| 2. | U72978.1 |  | 1996 |
| 3. | U72977.1 |  | 1996 |
| 4. | U72976.1 |  | 1996 |
| 5. | U72975.1 |  | 1996 |
| 6. | U72974.1 |  | 1996 |
| 7. | KT809294.1 | Brazil | 2018 |
| 8. | KT809293.1 | Brazil | 2018 |
| 9. | KT809292.1 | Brazil | 2018 |
| 10. | KT809291.1 | Brazil | 2018 |
| 11. | KT809290.1 | Brazil | 2018 |
| 12. | KT809289.1 | Brazil | 2018 |
| 13. | KT809288.1 | Brazil | 2018 |
| 14. | KT809287.1 | Brazil | 2018 |
| 15. | KT809286.1 | Brazil | 2018 |
| 16. | KT809285.1 | Brazil | 2018 |
| 17. | KT809284.1 | Brazil | 2018 |
| 18. | KT809283.1 | Brazil | 2018 |
| 19. | KT809282.1 | Brazil | 2018 |
| 20. | KT809281.1 | Brazil | 2018 |
| 21 | KT809280.1 | Brazil | 2018 |
| 22. | KT809279.1 | Brazil | 2018 |
| 23. | KT809278.1 | Brazil | 2018 |
| 24. | KT809277.1 | Brazil | 2018 |
| 25. | KT809276.1 | Brazil | 2018 |
| 26. | KT809275.1 | Brazil | 2018 |
| 27. | KT809274.1 | Brazil | 2018 |
| 28. | KT809273.1 | Brazil | 2018 |
| 29. | KT809272.1 | Brazil | 2018 |
| 30. | KT809271.1 | Brazil | 2018 |
| 31. | KT809270.1 | Brazil | 2018 |
| 32. | KT809269.1 | Brazil | 2018 |
| 33. | KT809268.1 | Brazil | 2018 |
| 34. | KT809267.1 | Brazil | 2018 |
| 35. | KT809266.1 | Brazil | 2018 |
| 36. | KT809265.1 | Brazil | 2018 |
| 37. | KT809264.1 | Brazil | 2018 |
| 38. | KT809263.1 | Brazil | 2018 |
| 39. | KT809262.1 | Brazil | 2018 |
| 40. | KT809261.1 | Brazil | 2018 |
| 41. | KT809260.1 | Brazil | 2018 |
| 42. | KT809259.1 | Brazil | 2018 |
| 43. | KT809258.1 | Brazil | 2018 |
| 44. | KT809257.1 | Brazil | 2018 |
| 45. | KT809256.1 | Brazil | 2018 |
| 46. | KT809255.1 | Brazil | 2018 |
| 47. | KT809254.1 | Brazil | 2018 |
| 48. | KT809253.1 | Brazil | 2018 |
| 49. | KT809252.1 | Brazil | 2018 |
| 50. | KT809251.1 | Brazil | 2018 |
| 51. | KT809250.1 | Brazil | 2018 |
| 52. | KT809249.1 | Brazil | 2018 |
| 53. | KT809248.1 | Brazil | 2018 |
| 54. | KT809247.1 | Brazil | 2018 |
| 55. | KT809246.1 | Brazil | 2018 |
| 56. | KT809245.1 | Brazil | 2018 |
| 57. | KT809244.1 | Brazil | 2018 |
| 58. | KT809243.1 | Brazil | 2018 |
| 59. | KT809242.1 | Brazil | 2018 |
| 60. | KT809241.1 | Brazil | 2018 |
| 61. | KT809240.1 | Brazil | 2018 |
| 62. | KT809239.1 | Brazil | 2018 |
| 63. | KT809238.1 | Brazil | 2018 |
| 64. | KT809237.1 | Brazil | 2018 |
| 65. | KT809236.1 | Brazil | 2018 |
| 66. | KT809235.1 | Brazil | 2018 |
| 67. | KJ634298.1 | Suriname | 2014 |
| 68. | KJ634297.1 | Honduras | 2014 |
| 69. | MK318422.1 | Mexico | 2019 |
| 70. | MK318420.1 | Mexico | 2019 |
| 71. | MK318377.1 | Puerto Rico | 2019 |
| 72. | MK318373.1 | Puerto Rico | 2019 |
| 73. | MK318372.1 | Mexico | 2019 |
| 74. | MK318311.1 | Mexico | 2019 |
| 75. | MK318297.1 | Dominican Republic | 2019 |
| 76. | GU439151.1 | Ontario | 2018 |
| 77. | GU439150.1 | Puslinch | 2018 |
| 78. | GU439149.1 | Puslinch | 2018 |
| 79. | GU439148.1 | Puslinch | 2018 |
| 80. | GU439147.1 | Puslinch | 2018 |
| 81. | GU090724.1 | Puslinch | 2018 |
| 82. | GU090723.1 | Puslinch | 2018 |
| 83. | GU095403.1 | New Brunswick | 2018 |
| 84. | GU094756.1 | Puslinch | 2018 |
| 85. | GU094755.1 | Puslinch | 2018 |
| 86. | GU094754.1 | Puslinch | 2018 |
| 87. | KJ388147.1 | Quebec | 2018 |
| 88. | HM102314.1 |  | 2016 |
| 89. | KJ641998.1 | Guano | 2015 |
| 90. | KJ641997.1 | Guano | 2015 |
| 91. | KF624877.1 | Roraima | 2014 |
| 92. | KF624876.1 | Roraima | 2014 |
| 93. | JQ559528.1 | Costa Rica | 2012 |
| 94. | JQ554012.1 | Costa Rica | 2012 |
| 95. | JQ572603.1 | Costa Rica | 2012 |
| 96. | JQ571459.1 | Costa Rica | 2012 |
| 97. | JQ547900.1 | Costa rica | 2012 |
| 98. | JQ577923.1 | Costa Rica | 2012 |
| 99. | JF854747.1 | Campina Grande | 2012 |
| 100. | JF854746.1 | Morretes | 2012 |
| 101. | JF854745.1 | Morretes | 2012 |
| 102. | JF854744.1 | Campina Grande | 2012 |
| 103. | JF854743.1 | Morretes | 2012 |
| 104. | JF854741.1 | Morretes | 2012 |
| 105. | JF854740.1 | Morretes | 2012 |
| 106. | HQ964527.1 | Massachusetts | 2012 |
| 107. | HQ964487.1 | Massachusetts | 2012 |
| 108. | HQ964486.1 | Massachusetts | 2012 |
| 109. | HQ964485.1 | Massachusetts | 2012 |
| 110. | HQ964443.1 | Massachusetts | 2012 |
| 111. | HQ964441.1 | Massachusetts | 2012 |
| 112. | HQ964442.1 | Massachusetts | 2012 |
| 113. | HQ964440.1 | Massachusetts | 2012 |
| 114. | HQ964439.1 | Massachusetts | 2012 |
| 115. | HQ964394.1 | Massachusetts | 2012 |
| 116. | HQ964393.1 | Massachusetts | 2012 |
| 117. | HQ964353.1 | Massachusetts | 2012 |
| 118. | HQ964352.1 | Massachusetts | 2012 |
| 119. | HQ964351.1 | Massachusetts | 2012 |
| 120. | GU159435.1 | Costa Rica | 2012 |
| 121. | GU159434.1 | Costa Rica | 2012 |
| 122. | GU159433.1 | Costa Rica | 2012 |
| 123. | GU159432.1 | Costa Rica | 2012 |
| 124. | GU159431.1 | Costa Rica | 2012 |
| 125. | GU159430.1 | Costa Rica | 2012 |
| 126. | GU159429.1 | Costa Rica | 2012 |
| 127. | GU159428.1 | Costa Rica | 2012 |
| 128. | GU159427.1 | Costa Rica | 2012 |
| 129. | GU159426.1 | Costa Rica | 2012 |
| 130. | GU163698.1 | Costa Rica | 2012 |
| 131. | HM136602.1 | Florida | 2012 |
| 132. | HM136601.1 | Florida | 2012 |
| 133. | HM136600.1 | Florida | 2012 |
| 134. | HM136599.1 | Florida | 2012 |
| 135. | HM136598.1 | Florida | 2012 |
| 136. | HM136597.1 | Florida | 2012 |
| 137. | HM136596.1 | Florida | 2012 |
| 138. | HM136595.1 | Florida | 2012 |
| 139. | HM136594.1 | Florida | 2012 |
| 140. | HM136593.1 | Florida | 2012 |
| 141. | HM136592.1 | Florida | 2012 |
| 142. | HM136591.1 | Florida | 2012 |
| 143. | HM136590.1 | Florida | 2012 |
| 144. | HM136589.1 | Florida | 2012 |
| 145. | HM136588.1 | Florida | 2012 |
| 146. | HM136587.1 | Florida | 2012 |
| 147. | HM136586.1 | Florida | 2012 |
| 148. | HM388081.1 | Bartlesville | 2019 |
| 149. | GU799699.1 | Bartlesville | 2019 |
| 150. | MG360803.1 | Ontario | 2018 |
| 151. | GU658451.1 | Alvaro Obregon | 2019 |
| 152. | JF857952.1 | Yucatan | 2019 |
| 153. | JF855010.1 | North Carolina | 2019 |
| 154. | HM406395.1 | Arizona | 2019 |
| 155. | GU669423.1 | Maryland | 2019 |
| 156. | HM406394.1 | Arizona | 2019 |
| 157. | JF855008.1 | Bryson City | 2019 |
| 158. | HM430360.1 | Arizona | 2019 |
| 159. | KJ381264.1 | Ontario | 2018 |
| 160. | KJ393860.1 | Ontario | 2018 |
| 161. | MG360372.1 | Ontario | 2018 |
| 162. | KJ389856.1 | Quebec | 2018 |
| 163. | KF854210.1 |  | 2015 |

C) COIA gene sequences from Africa

| S.No. | GenBank Accession | Location | Year  Submitted |
| --- | --- | --- | --- |
| 1. | MF593258.1 | South Africa | 2018 |
| 2. | MF593257.1 | South Africa | 2018 |
| 3. | MF593256.1 | South Africa | 2018 |
| 4. | MF593255.1 | South Africa | 2018 |
| 5. | MF593254.1 | South Africa | 2018 |
| 6. | MF593253.1 | South Africa | 2018 |
| 7. | MF593252.1 | South Africa | 2018 |
| 8. | MF593251.1 | South Africa | 2018 |
| 9. | MF593250.1 | South Africa | 2018 |
| 10. | MF593249.1 | South Africa | 2018 |
| 11. | MF593248.1 | South Africa | 2018 |
| 12. | MF593247.1 | South Africa | 2018 |
| 13. | MF593246.1 | South Africa | 2018 |
| 14. | MF593245.1 | South Africa | 2018 |
| 15. | MF593244.1 | South Africa | 2018 |
| 16. | MF593243.1 | South Africa | 2018 |
| 17. | MF593242.1 | South Africa | 2018 |
| 18. | MF593241.1 | South Africa | 2018 |
| 19. | MK493022.1 | NW Province South Africa | 2019 |
| 20. | MK493021.1 | NW Province South Africa | 2019 |
| 21 | MK493020.1 | NW Province South Africa | 2019 |
| 22. | MK493019.1 | NW Province South Africa | 2019 |
| 23. | MK493018.1 | NW Province South Africa | 2019 |
| 24. | MK493017.1 | NW Province South Africa | 2019 |
| 25. | MK493016.1 | NW Province South Africa | 2019 |
| 26. | MK493015.1 | NW Province South Africa | 2019 |
| 27. | MK493014.1 | NW Province South Africa | 2019 |
| 28. | MK493013.1 | NW Province South Africa | 2019 |
| 29. | MK493012.1 | NW Province South Africa | 2019 |
| 30. | MK493011.1 | NW Province South Africa | 2019 |
| 31. | MT103351.1 | Congo | 2020 |
| 32. | MT103350.1 | Congo | 2020 |
| 33. | MT103349.1 | Congo | 2020 |
| 34. | MT103348.1 | Tanzania | 2020 |
| 35. | MT103347.1 | Mazowe | 2020 |
| 36. | MT103346.1 | Harare | 2020 |
| 37. | KX580619.1 | Nigeria | 2016 |
| 38. | KX580618.1 | Nigeria | 2016 |
| 39. | KX580617.1 | Nigeria | 2016 |
| 40. | KX580616.1 | Nigeria | 2016 |
| 41. | KX580615.1 | Sao-Tome, Porto Allegre | 2016 |
| 42. | KX580614.1 | Sao-Tome | 2016 |
| 43. | MT641267.1 | Uganda | 2020 |
| 44. | MF278659.1 | Tanzania | 2018 |
| 45. | MF278658.1 | Tanzania | 2018 |
| 46. | MF278657.1 | Tanzania | 2018 |
| 47. | MH190448.1 | Kenya | 2018 |
| 48. | MH190447.1 | Kenya | 2018 |
| 49. | MH190446.1 | Kenya | 2018 |
| 50. | MH190445.1 | Kenya | 2018 |
| 51. | MH190444.1 | Kenya | 2018 |
| 52. | KY472255.1 | Ghana: Volta region | 2017 |
| 53. | KY472254.1 | Ghana: Volta region | 2017 |
| 54. | KY472253.1 | Ghana: Volta region | 2017 |
| 55. | KY472252.1 | Ghana: Volta region | 2017 |
| 56. | KY472251.1 | Ghana: Volta region | 2017 |
| 57. | KY472250.1 | Ghana: Northern region | 2017 |
| 58. | KY472249.1 | Ghana: Northern region | 2017 |
| 59. | KY472248.1 | Ghana: Northern region | 2017 |
| 60. | KY472245.1 | Ghana | 2017 |
| 61. | KY472244.1 | Ghana | 2017 |
| 62. | KY472242.1 | Ghana: Brong Ahafo | 2017 |
| 63. | KY472241.1 | Ghana: Brong Ahafo | 2017 |
| 64. | KY472240.1 | Ghana: Brong Ahafo | 2017 |
| 65. | MG993205.1 | Malawi: Sande | 2018 |
| 66. | MF197867.1 | Uganda | 2018 |
| 67. | MK493006.1 | Kenya: Taita Taveta County | 2019 |
| 68. | MK493000.1 | Kenya: Taita Taveta County | 2019 |
| 69. | MK492996.1 | Kenya: Kwale County | 2019 |
| 70. | MK493010.1 | Kenya: Taita Taveta County | 2019 |
| 71. | MK493009.1 | Kenya: Taita Taveta County | 2019 |
| 72. | MK493008.1 | Kenya: Taita Taveta County | 2019 |
| 73. | MK493007.1 | Kenya: Taita Taveta County | 2019 |
| 74. | MK493004.1 | Kenya: Taita Taveta County | 2019 |
| 75. | MK493003.1 | Kenya: Taita Taveta County | 2019 |
| 76. | MK493002.1 | Kenya: Taita Taveta County | 2019 |
| 77. | MK493001.1 | Kenya: Taita Taveta County | 2019 |
| 78. | MK492999.1 | Kenya: Taita Taveta County | 2019 |
| 79. | MK492998.1 | Kenya: Taita Taveta County | 2019 |
| 80. | MK492997.1 | Kenya: Kwale County | 2019 |
| 81. | MK492995.1 | Kenya: Kwale County | 2019 |
| 82. | MK492994.1 | Kenya: Kwale County | 2019 |
| 83. | MK492993.1 | Kenya: Kwale County | 2019 |
| 84. | MK492992.1 | Kenya: Kwale County | 2019 |
| 85. | MK492991.1 | Kenya: Kwale County | 2019 |
| 86. | MK492990.1 | Kenya: Kwale County | 2019 |
| 87. | MK492989.1 | Kenya: Kwale County | 2019 |
| 88. | MK492988.1 | Kenya: Kwale County | 2019 |
| 89. | MK492987.1 | Kenya: Kwale County | 2019 |
| 90. | MK492986.1 | Kenya: Kwale County | 2019 |
| 91. | MK492985.1 | Kenya: Kwale County | 2019 |
| 92. | MK492984.1 | Kenya: Kwale County | 2019 |
| 93. | MK492983.1 | Kenya: Kwale County | 2019 |
| 94. | MK492982.1 | Kenya: Kwale County | 2019 |
| 95. | MK492981.1 | Kenya: Kwale County | 2019 |
| 96. | MK492979.1 | Kenya: Kwale County | 2019 |
| 97. | MK492978.1 | Kenya: Kwale County | 2019 |
| 98. | MK492977.1 | Kenya: Kwale County | 2019 |
| 99. | MK492976.1 | Kenya: Kwale County | 2019 |
| 100. | MK492975.1 | Kenya: Kwale County | 2019 |
| 101. | MK492973.1 | Kenya: Kwale County | 2019 |
| 102. | MK492972.1 | Kenya: Kwale County | 2019 |
| 103. | MK492971.1 | Kenya: Kwale County | 2019 |
| 104. | MK492970.1 | Kenya: Kwale County | 2019 |
| 105. | MK492969.1 | Kenya: Kwale County | 2019 |
| 106. | MK492968.1 | Kenya: Kwale County | 2019 |
| 107. | MK492967.1 | Kenya: Kwale County | 2019 |
| 108. | MK492966.1 | Kenya: Kwale County | 2019 |
| 109. | MK492965.1 | Kenya: Kwale County | 2019 |
| 110. | MK492964.1 | Kenya: Kwale County | 2019 |
| 111. | MK492963.1 | Kenya: Kwale County | 2019 |
| 112. | MK492962.1 | Kenya: Trans Nzoia County | 2019 |
| 113. | MK492961.1 | Kenya: Trans Nzoia County | 2019 |
| 114. | MK492960.1 | Kenya: Trans Nzoia County | 2019 |
| 115. | MK492959.1 | Kenya: Trans Nzoia County | 2019 |
| 116. | MK492958.1 | Kenya: Trans Nzoia County | 2019 |
| 117. | MK492957.1 | Kenya: Trans Nzoia County | 2019 |
| 118. | MK492956.1 | Kenya: Trans Nzoia County | 2019 |
| 119. | MK492955.1 | Kenya: Trans Nzoia County | 2019 |
| 120. | MK492954.1 | Kenya: Trans Nzoia County | 2019 |
| 121. | MK492953.1 | Kenya: Trans Nzoia County | 2019 |
| 122. | MK492952.1 | Kenya: Trans Nzoia County | 2019 |
| 123. | MK492951.1 | Kenya: Uasin Gishu County | 2019 |
| 124. | MK492950.1 | Kenya: Uasin Gishu County | 2019 |
| 125. | MK492949.1 | Kenya: Uasin Gishu County | 2019 |
| 126. | MK492948.1 | Kenya: Uasin Gishu County | 2019 |
| 127. | MK492947.1 | Kenya: Uasin Gishu County | 2019 |
| 128. | MK492946.1 | Kenya: Uasin Gishu County | 2019 |
| 129. | MK492945.1 | Kenya: Uasin Gishu County | 2019 |
| 130. | MK492944.1 | Kenya: Uasin Gishu County | 2019 |
| 131. | MK492943.1 | Kenya: Uasin Gishu County | 2019 |
| 132. | MK492942.1 | Kenya: Uasin Gishu County | 2019 |
| 133. | MK492939.1 | Kenya: Uasin Gishu County | 2019 |
| 134. | MK492938.1 | Kenya: Uasin Gishu County | 2019 |
| 135. | MK492936.1 | Kenya: Uasin Gishu County | 2019 |
| 136. | MK492933.1 | Kenya: Uasin Gishu County | 2019 |
| 137. | MK492930.1 | Kenya: Uasin Gishu County | 2019 |
| 138. | MK492929.1 | Kenya: Uasin Gishu County | 2019 |
| 139. | MF197868.1 | Uganda | 2018 |
| 140. | MK492980.1 | Kenya: Kwale County | 2019 |
| 141. | MK492941.1 | Kenya: Uasin Gishu County | 2019 |
| 142. | MK492940.1 | Kenya: Uasin Gishu County | 2019 |
| 143. | MK492937.1 | Kenya: Uasin Gishu County | 2019 |
| 144. | MK492935.1 | Kenya: Uasin Gishu County | 2019 |
| 145. | MK492934.1 | Kenya: Uasin Gishu County | 2019 |
| 146. | MK492932.1 | Kenya: Uasin Gishu County | 2019 |
| 147. | MK492931.1 | Kenya: Uasin Gishu County | 2019 |
| 148. | MK493005.1 | Kenya: Taita Taveta County | 2019 |
| 149. | MK492974.1 | Kenya: Kwale County | 2019 |
| 150. | MT933057.1 | Tanzania | 2020 |

D) COIA gene sequences from Asia-II

| S.No. | GenBank Accession | Location | Year  Submitted |
| --- | --- | --- | --- |
| 1. | MT103344.1 | Bangladesh: Dhaka | 2020 |
| 2. | MT103343.1 | Bangladesh: Dhaka | 2020 |
| 3. | MT103342.1 | South Korea: Gyeongsan | 2020 |
| 4. | MT103341.1 | Viet Nam: Ninh binh | 2020 |
| 5. | MT103340.1 | Viet Nam: Ninh binh | 2020 |
| 6. | MT103339.1 | Viet Nam: Ha noi | 2020 |
| 7. | MT103338.1 | Viet Nam: Vinh phuc | 2020 |
| 8. | MT103336.1 | Viet Nam: Hanoi | 2020 |
| 9. | MT103335.1 | Viet Nam: Vinh Phuc | 2020 |
| 10. | MT103334.1 | Viet Nam: Ninh Binh | 2020 |
| 11. | MT641270.1 | South Korea: Gyeongsan | 2020 |
| 12. | MT641269.1 | South Korea: Jeju | 2020 |
| 13. | MT641268.1 | South Korea: Campus | 2020 |
| 14. | LC546868.1 | Japan: Aomori | 2020 |
| 15. | LC546867.1 | Japan: Aomori | 2020 |
| 16. | LC546866.1 | Japan: Iwate | 2020 |
| 17. | LC546865.1 | Japan: Kanagawa | 2020 |
| 18. | LC546864.1 | Japan: Chiba | 2020 |
| 19. | LC546863.1 | Japan: Fukushima | 2020 |
| 20. | LC546862.1 | Japan: Ibaraki | 2020 |
| 21 | LC546861.1 | Japan: Ibaraki | 2020 |
| 22. | LC546860.1 | Japan: Miyazaki | 2020 |
| 23. | LC546859.1 | Japan: Miyazaki | 2020 |
| 24. | LC546858.1 | Japan: Miyazaki | 2020 |
| 25. | LC546857.1 | Japan: Okinawa | 2020 |
| 26. | LC546856.1 | Japan: Okinawa | 2020 |
| 27. | LC546855.1 | Japan: Okinawa | 2020 |
| 28. | LC546854.1 | Japan: Kagoshima | 2020 |
| 29. | LC546853.1 | Japan: Kagoshima | 2020 |
| 30. | LC546852.1 | Japan: Kagoshima | 2020 |
| 31. | LC546851.1 | Japan: Kagoshima | 2020 |
| 32. | LC546850.1 | Japan: Kagoshima | 2020 |
| 33. | LC546849.1 | Japan: Kagoshima | 2020 |
| 34. | LC546848.1 | Japan: Kagoshima | 2020 |
| 35. | LC546847.1 | Japan: Kagoshima | 2020 |
| 36. | LC546846.1 | Japan: Kagoshima | 2020 |
| 37. | MK913648.1 | Viet Nam: Nghe An | 2019 |
| 38. | MK913647.1 | Viet Nam: Nghe An | 2019 |
| 39. | MK913646.1 | Viet Nam: Ha Noi | 2019 |
| 40. | MK860942.1 | China: Tengchong, Yunnan | 2019 |
| 41. | MK860941.1 | China: Tengchong, Yunnan | 2019 |
| 42. | MK860940.1 | China: Tengchong, Yunnan | 2019 |
| 43. | MK860939.1 | China: Tengchong, Yunnan | 2019 |
| 44. | MK860938.1 | China: Tengchong, Yunnan | 2019 |
| 45. | MK860937.1 | China: Tengchong, Yunnan | 2019 |
| 46. | MK860936.1 | China: Ruili, Yunnan | 2019 |
| 47. | MK860935.1 | China: Ruili, Yunnan | 2019 |
| 48. | MK860934.1 | China: Ruili, Yunnan | 2019 |
| 49. | MK860933.1 | China: Ruili, Yunnan | 2019 |
| 50. | MK860932.1 | China: Ruili, Yunnan | 2019 |
| 51. | MK860931.1 | China: Ruili, Yunnan | 2019 |
| 52. | MK860930.1 | China: Ruili, Yunnan | 2019 |
| 53. | MK860929.1 | China: Ruili, Yunnan | 2019 |
| 54. | MK860928.1 | China: Ruili, Yunnan | 2019 |
| 55. | MK860927.1 | China: Ruili, Yunnan | 2019 |
| 56. | MK860926.1 | China: Ruili, Yunnan | 2019 |
| 57. | MK860925.1 | China: Ruili, Yunnan | 2019 |
| 58. | MK860924.1 | China: Ruili, Yunnan | 2019 |
| 59. | MK860923.1 | China: Mangshi, Yunnan | 2019 |
| 60. | MK860922.1 | China: Mangshi, Yunnan | 2019 |
| 61. | MK860921.1 | China: Mangshi, Yunnan | 2019 |
| 62. | MK860920.1 | China: Mangshi, Yunnan | 2019 |
| 63. | MK860919.1 | China: Mangshi, Yunnan | 2019 |
| 64. | MK860918.1 | China: Mangshi, Yunnan | 2019 |
| 65. | MK713974.1 | Myanmar: SR4, Eastern Shan State | 2019 |
| 66. | MN075831.1 | China | 2019 |
| 67. | MN075830.1 | China | 2019 |
| 68. | MK913645.1 | Viet Nam: Ninh Binh | 2019 |
| 69. | MT073263.1 | Bangladesh: Gazipur | 2020 |
| 70. | MT180097.1 | Pakistan | 2020 |
| 71. | MK790611.1 | China | 2019 |
| 72. | MT073264.1 | Bangladesh: Bogura | 2020 |
| 73. | MT073266.1 | Bangladesh: Jamalpur | 2020 |
| 74. | MT073265.1 | Bangladesh: Rangpur | 2020 |
| 75. | MN820655.1 | China | 2019 |
| 76. | MN820654.1 | China | 2019 |

Table 2: List of *S. frugiperda* COIB gene sequences used in the study.

| S.No. | GenBank Accession | Location |
| --- | --- | --- |
| 1. | MW287338 | NagarkurnoolTelangana |
| 2. | MW287339 | Khammam, Telangana |
| 3. | MW287340 | Nelivada, Andhra Pradesh |
| 4. | MW287341 |  |
| 5. | MW287342 | Chintapalli, Andhra Pradesh |
| 6. | MW287343 | Anakapalle, Andhra Pradesh |
| 7. | MW279226 | Tirupati |
| 8. | MW279227 | Malada, West Bengal |
| 9. | MW279228 | Hassan |
| 10. | MW279229 | Belur |
| 11. | MW279230 | Shimoga |
| 12. | MW279231 | Siddipet |
| 13. | MW408114 | Dharwad, Karnataka |
| 14. | MW408115 | Vijayawada, Andhra Pradesh |
| 15. | MW408116 | Nepal |
| 16. | MW408117 | Khumaltar,Nepal |
| 17. | MW408118 | Nawalpur, Nepal |
| 18. | MW408119 | Nepal |
| 19. | MW408120 | Chikkaballapur, Karnataka |
| 20. | MW408121 | westbengal |
| 21 | MW455100 | Kerala |
| 22. | MW455101 | Tripura |
| 23. | MW455102 | Manipur |
| 24. | MW455103 | Mizoram |
| 25. | MW456329 | Punjab, Ludhiana |
| 26. | MW456330 | Punjab, Ludhiana |
| 27. | MW456331 | Punjab, Ludhiana |
| 28. | MW456332 | Punjab, Ludhiana |
| 29. | MW457628 | Coimbatore, TN |
| 30. | MW457629 | Kolarpettai, TN |
| 31. | MW457630 | Udumalaipettai, TN |
| 32. | MW457631 | Sathnur, TN |
| 33. | MW457627 | Tamilnadu |
| 34. | MW465232 | Chickballapur |
| 35. | MW465233 | Dharmapuri |
| 36. | MW465234 | Ananthpur |
| 37. | MW465235 | Attur |
| 38. | MW465236 | Gauribidanur |
| 39. | MW484885 | Pune |
| 40. | MW484886 | Bagalkot |
| 41. | MW484883 | Tirupati2 |
| 42. | MW564033 | Pennagaram, Tamilnadu |
| 43. | MW564034 | Pennagaram, Tamilnadu |
| 44. | MW564035 | Pennagaram, Tamilnadu |
| 45. | MW564036 | Pennagaram, Tamilnadu |
| 46. | MW564037 | Pennagaram, Tamilnadu |
| 47. | MW564038 | Pennagaram, Tamilnadu |
| 48. | MW564039 | Pennagaram, Tamilnadu |
| 49. | MW564040 | Pennagaram, Tamilnadu |
| 50. | MW564041 | Pennagaram, Tamilnadu |
| 51. | MW564042 | Pennagaram, Tamilnadu |
| 52. | MW584913 | Raichur, Karnataka |
| 53. | MW584914 | Raichur, Karnataka |
| 54. | MW564043 | Raichur, Karnataka |
| 55. | MW564044 | Raichur, Karnataka |
| 56. | MW564045 | Raichur, Karnataka |
| 57. | MW564046 | Raichur, Karnataka |
| 58. | MW564047 | Raichur, Karnataka |
| 59. | MW564048 | Raichur, Karnataka |
| 60. | MW584915 | Raichur, Karnataka |
| 61. | MW584916 | Raichur, Karnataka |
| 62. | MW564049 | Parbhani, Maharashtra |
| 63. | MW584917 | Parbhani, Maharashtra |
| 64. | MW584918 | Parbhani, Maharashtra |
| 65. | MW584919 | Parbhani, Maharashtra |
| 66. | MW584920 | Parbhani, Maharashtra |
| 67. | MW584921 | Parbhani, Maharashtra |
| 68. | MW584922 | Parbhani, Maharashtra |
| 69. | MW584923 | Chickballapur |
| 70. | MW564050 | Kolar |
| 71. | MW564051 | Kolar |
| 72. | MW564052 | Doddaballapur |
| 73. | MW564053 | Kanakapura |

Table 3: List of *S. frugiperda* *Tpi* gene sequences used in the study.

| S.No. | GenBank Accession | Location |
| --- | --- | --- |
| 1. | MW468070 | Khumaltar,Nepal |
| 2. | MW468071 | Nepal |
| 3. | MW468072 | Nawalpur, Nepal |
| 4. | MW468073 | Chintapalli, Andhra Pradesh |
| 5. | MW468074 | Tirupati |
| 6. | MW468075 | Pune Maharastra |
| 7. | MW468076 | Shimoga |
| 8. | MW468077 | Malada, West Bengal |
| 9. | MW468078 | Chikkaballapur, Karnataka |
| 10. | MW468079 | Nelivada, Andhra Pradesh |
| 11. | MW468080 | Rajendranagar Telangana |
| 12. | MW468081 | Meghalaya |
| 13. | MW468082 | Anakapalle, Andhra Pradesh |
| 14. | MW468083 | Bagalkot karnataka |
| 15. | MW468084 | Dodaballapur Karnataka |
| 16. | MW468085 | NagarkurnoolTelangana |
| 17. | MW468086 | Siddipet |
| 18. | MW582637 | Pennagaram, Tamilnadu |
| 19. | MW582638 | Pennagaram, Tamilnadu |
| 20. | MW582639 | Pennagaram, Tamilnadu |
| 21 | MW582640 | Pennagaram, Tamilnadu |
| 22. | MW582641 | Pennagaram, Tamilnadu |
| 23. | MW582642 | Pennagaram, Tamilnadu |
| 24. | MW582643 | Pennagaram, Tamilnadu |
| 25. | MW582644 | Pennagaram, Tamilnadu |
| 26. | MW582645 | Pennagaram, Tamilnadu |
| 27. | MW582646 | Pennagaram, Tamilnadu |
| 28. | MW582647 | Raichur, Karnataka |
| 29. | MW582648 | Raichur, Karnataka |
| 30. | MW582649 | Raichur, Karnataka |
| 31. | MW582650 | Raichur, Karnataka |
| 32. | MW582651 | Raichur, Karnataka |
| 33. | MW583570 | Raichur, Karnataka |
| 34. | MW583571 | Raichur, Karnataka |
| 35. | MW583572 | Raichur, Karnataka |
| 36. | MW583573 | Raichur, Karnataka |
| 37. | MW583574 | Raichur, Karnataka |
| 38. | MW583575 | Parbhani, Maharashtra |
| 39. | MW583576 | Parbhani, Maharashtra |
| 40. | MW583577 | Parbhani, Maharashtra |
| 41. | MW583578 | Parbhani, Maharashtra |
| 42. | MW583579 | Parbhani, Maharashtra |
| 43. | MW583580 | Parbhani, Maharashtra |
| 44. | MW583581 | Parbhani, Maharashtra |
| 45. | MW583582 | Parbhani, Maharashtra |
| 46. | MW583583 | Parbhani, Maharashtra |
| 47. | MW583584 | Chickballapur |
| 48. | MW583585 | Kolar |
| 49. | MW583586 | Kolar |
| 50. | MW583587 | Doddaballapur |
| 51. | MW583588 | Kanakapura |
| 52. | MW493651 | Tripura |
| 53. | MW493653 | Manipur |
| 54. | MW493654 | Mizoram |
| 55. | MW493660 | Punjab, Ludhiana |
| 56. | MW493661 | Punjab, Ludhiana |
| 57. | MW493662 | Punjab, Ludhiana |
| 58. | MW493663 | Punjab, Ludhiana |
| 59. | MW493650 | Kolarpettai, TN |
| 60. | MW517579 | Udumalaipettai, TN |
| 61. | MW493649 | Sathnur, TN |
| 62. | MW493655 | Chickballapur |
| 63. | MW493656 | Dharmapuri |
| 64. | MW493657 | Ananthpur |
| 65. | MW493658 | Attur |
| 66. | MW493659 | Gauribidanur |

Table 4: Summary of the genetic diversity of mtCOIA gene of Indian FAW populations from primary data generated at ICAR-NBAIR

| No. of sequences | 92 |
| --- | --- |
| No. of sites | 459 |
| No. of polymorphic sites | 6 |
| No. of mutations | 6 |
| No. of haplotypes | 2 |
| Haplotype diversity | 0.104 |
| Nucleotide diversity | 0.00136 |
| Fu's Fs statistic | 2.872 |
| Fu and Li's D* test statistic | 1.12 |
| Fu and Li's F* test statistic | 0.452 |
| Tajima's D | -1.07 |
